# Dominantly inherited muscle disorders: understanding their complexity and exploring therapeutic approaches

**DOI:** 10.1242/dmm.050720

**Published:** 2024-11-06

**Authors:** Andrew R. Findlay

**Affiliations:** Washington University Saint Louis, Neuromuscular Disease Center, 660 S. Euclid Ave., St Louis, MO 63110, USA

**Keywords:** Dominant disease mechanisms, Dominant inheritance, Muscular dystrophy, Myopathy

## Abstract

Treatments for disabling and life-threatening hereditary muscle disorders are finally close to becoming a reality. Research has thus far focused primarily on recessive forms of muscle disease. The gene replacement strategies that are commonly employed for recessive, loss-of-function disorders are not readily translatable to most dominant myopathies owing to the presence of a normal chromosome in each nucleus, hindering the development of novel treatments for these dominant disorders. This is largely due to their complex, heterogeneous disease mechanisms that require unique therapeutic approaches. However, as viral and RNA interference-based therapies enter clinical use, key tools are now in place to develop treatments for dominantly inherited disorders of muscle. This article will review what is known about dominantly inherited disorders of muscle, specifically their genetic basis, how mutations lead to disease, and the pathomechanistic implications for therapeutic approaches.

## Teaser

**Summary:** Dominantly inherited myopathies and muscular dystrophies affect various skeletal muscle functions, from structural components to protein homeostasis. Understanding their complex, diverse mechanisms is crucial for developing effective therapies.

## Introduction

**Fig. 1.**
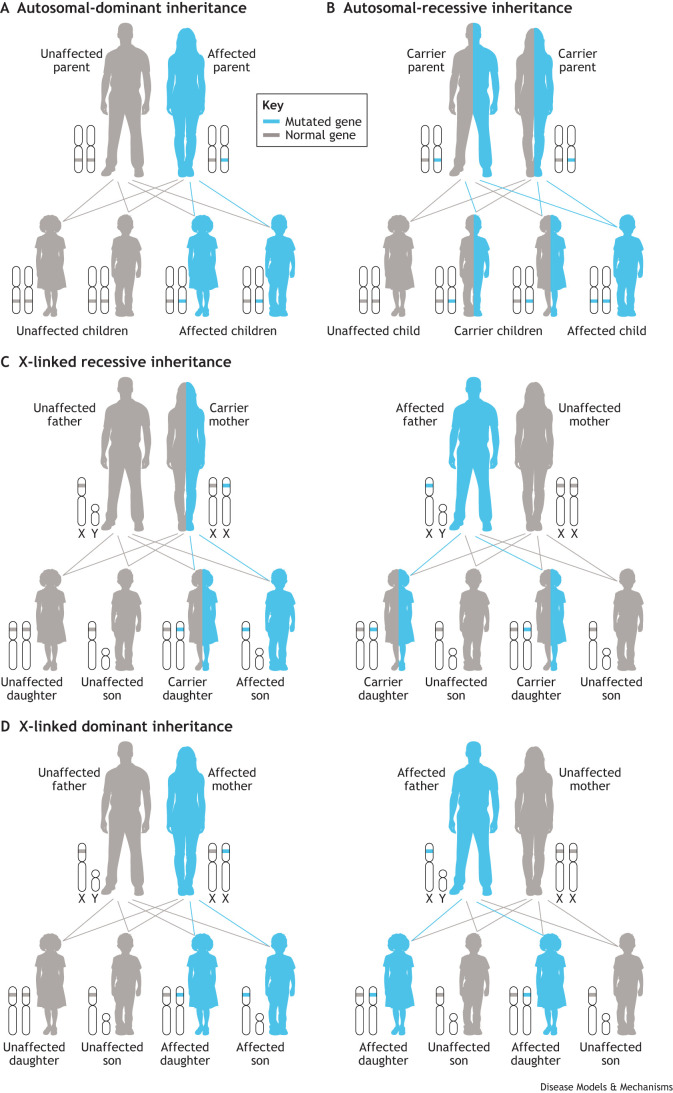
**Patterns of inheritance of skeletal muscle disorders.** (A) Autosomal dominant. (B) Autosomal recessive. (C) X-linked recessive. (D) X-linked dominant.

Dominantly inherited muscle disorders are a group of genetic conditions that affect skeletal muscle. In autosomal-dominant disorders, an affected individual has a 50% chance of passing the mutated gene on to their offspring (Fig. 1A). These disorders typically lead to progressive muscle weakness affecting various muscle groups in the body, including skeletal muscles and sometimes cardiac muscles. A combined prevalence rate of all dominantly inherited myopathies has not been determined. For individual disorders, there is a wide range of regionally variable prevalence, with facioscapulohumeral muscular dystrophy (FSHD) (0.22-6.66 per 100,000) (Flanigan et al., 2001; Padberg, 1982) and myotonic dystrophy type 1 (0.37-35 per 100,000) (Liao et al., 2022) being among the most common. For all limb-girdle muscular dystrophies (LGMDs) combined (recessive and dominant) a prevalence of 1.63-2.27 per 100,000 has been estimated (Mah et al., 2016; Norwood et al., 2009), and dominant LGMDs have been estimated to account for ∼6-16% of all LGMDs (Magri et al., 2017; Nallamilli et al., 2018).

**Fig. 2.**
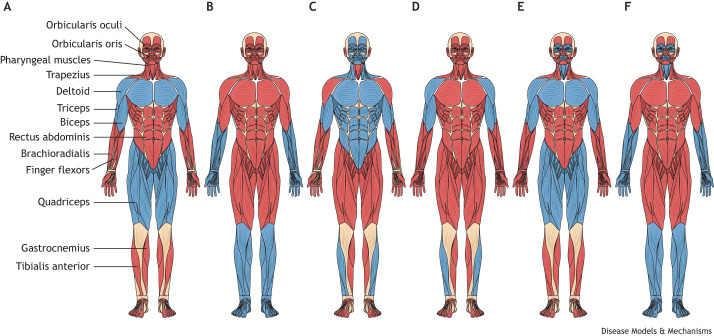
**Patterns of muscle weakness encountered in dominantly inherited disorders of muscle.** Red indicates normal muscles; blue indicates muscles that are weak. (A) Proximal weakness, as seen in limb-girdle muscular dystrophies (LGMDs). (B) Distal weakness, as seen in many myofibrillar myopathies. (C) Weakness seen in fascioscapulohumeral muscular dystrophy (FSHD). (D) Humeroperoneal pattern of weakness often seen in Emery-Dreifuss muscular dystrophies (EDMDs). (E) Weakness pattern seen in oculopharyngeal muscular dystrophy (OPMD). (F) Weakness pattern seen in oculopharyngeal distal myopathy (OPDM).

Some broad, but not universal, generalizations can be made when comparing dominant to recessive disorders (Fig. 1B,C). Dominant disorders of muscle can manifest at any age, ranging from infancy to late adulthood, whereas recessive disorders commonly present in childhood or early adulthood. Furthermore, the pattern of muscle weakness in dominant disorders may have a wider variation [distal, proximal scapuloperoneal (see Glossary, Box 1), etc.], whereas recessive disorders typically cause symmetric weakness that is either diffuse or proximal predominant (Fig. 2A-F). Dominant disorders have a wide range of prognoses, but many progress more slowly, and, in the absence of cardiac or respiratory involvement, they are often less severe than many recessively inherited myopathies. Diagnosis typically involves a combination of clinical assessment, genetic testing and sometimes muscle biopsies. Muscle pathology in dominant disorders again varies depending on the specific genetic cause. Most disorders typically cause myopathic changes (fiber size variability, rounded atrophic fibers, increased internal nuclei, etc.). Many also display evidence of protein aggregates, vacuoles and myofibrillar abnormalities. Although there are currently no cures for any dominantly inherited muscle disorders, management involves physical therapy, mobility aids and symptomatic treatments to compensate for muscle weakness and associated symptoms (Hamel, 2022; Johnson and Statland, 2022; Mul, 2022). For autosomal-dominant and X-linked disorders (Fig. 1A,D), it is especially crucial to provide appropriate genetic counseling to affected patients and family members about the 50% risk of passing on the disease to children (Basta and Pandya, 2024; Kingston, 1989).

**Box 1. Glossary**

**Adeno-associated virus (AAV):** a non-pathogenic virus used as a vector to deliver genetic material into cells. AAV vectors are commonly used in gene therapy owing to their ability to infect a wide range of cell types and induce long-lasting gene expression.

**Allele:** one of two or more versions of a DNA sequence (a single base or a segment of bases) at a given genomic location. An individual inherits two alleles, one from each parent, for any given genomic location in which such variation exists.

**Allele-specific:** refers to therapies or approaches that specifically target the mutant allele of a gene while sparing the normal allele. This is crucial in diseases in which the non-mutant allele is essential for normal function.

**Antisense oligonucleotide (ASO):** short, synthetic strands of nucleotides designed to specifically bind to the RNA transcripts of genes. ASOs can alter RNA splicing, block protein translation or induce the degradation of specific RNA molecules.

**Autophagic vacuoles with sarcolemmal features (AVSF):** specialized structures observed in muscle pathology, characterized by vacuoles that are lined by proteins typically found at the sarcolemma (muscle membrane). These vacuoles are involved in autophagy, and their presence can indicate disorders with disrupted autophagy or membrane integrity.

**Autophagolysosomal (APL) system:** a cellular degradation pathway that eliminates damaged organelles and proteins through lysosomal degradation.

**Axial musculature:** muscles that are located along the axis (central line) of the body. These include the muscles of the head, neck, spine and trunk. Axial muscles are essential for maintaining posture, supporting the spine, and allowing movements such as bending, twisting and turning.

**Axial rigidity:** stiffness and reduced flexibility of the spine and trunk muscles. Can be due to progressive muscle weakness and degeneration associated with various muscular dystrophies and myopathies, particularly those that affect the muscles supporting the body's core and spine.

**Bulbar musculature:** the group of muscles that are innervated by the cranial nerves, which emerge from the brainstem. These muscles primarily involve those responsible for facial expression, eye movement, chewing, swallowing and speech.

**Chaperone:** a type of protein that assists in the proper folding of other proteins, preventing misfolding and aggregation. They are pivotal in maintaining protein homeostasis within cells.

**Chaperone-assisted selective autophagy (CASA) pathway:** a specialized cellular pathway that mediates the selective degradation of cytoskeletal components under stress conditions. The CASA pathway involves chaperones, such as HSPB8 and BAG3, which identify and bind to damaged or misfolded proteins within the cell. These chaperone proteins then guide the tagged proteins to the autophagic machinery for degradation.

**Chaperonopathies:** diseases caused by mutations in chaperone proteins, leading to a wide spectrum of clinical manifestations owing to the disruption of protein quality control mechanisms in the cell.

**Chorioallantoic fusion:** the embryological process by which the chorion, the outermost membrane that surrounds the embryo, and the allantois, an embryonic outpouching of the yolk sac, come together to contribute to the development of the placenta. This fusion allows the blood vessels from the allantois, which extend into the umbilical cord, to integrate with the chorion. This integration is crucial for forming the vascular network within the placenta, facilitating effective exchange of nutrients, gases and wastes between the mother and the fetus.

**Chromatin:** complex of DNA and proteins, mainly histones. Chromatin's structure determines the accessibility of DNA to enzymes and other proteins that regulate gene expression and DNA replication.

**CRISPR/Cas9:** a gene-editing technology that can precisely modify DNA within organisms. It allows for the addition, removal or alteration of specific DNA sequences in the genome.

**Cytoskeleton:** a network of proteins including intermediate filaments and microtubules in the cytoplasm that provides structural support, facilitates cell movement, and directs organelle and molecular transport within the cell.

**Dominant negative:** a type of mutation where the mutant gene product interferes with the function of the normal gene product, often through forming non-functional complexes, leading to a loss of function in heterozygous cells. This is often seen with proteins that multimerize, where each protein complex that the mutant protein is a part of is rendered non-functional. For example, if a protein of interest forms a dimer, and if equal amounts of normal and mutant proteins are assumed, only 25% of dimers would be expected to be normal, and the remaining 75% would be non-functional owing to the presence of at least one mutant protein.

**Dysphagia:** medical term referring to difficulty swallowing.

**Dystrophin-glycoprotein complex (DGC):** a complex of proteins that connects the cytoskeleton of a muscle fiber to the surrounding extracellular matrix, crucial for the structural stability of muscle cells during contraction.

**Epigenetic repression:** the process by which gene activity is reduced or silenced without changing the DNA sequence, often through modifications to the chromatin structure.

**Excitation-contraction coupling:** the process by which an electrical impulse in a muscle cell leads to contraction. It involves the conversion of an action potential to a mechanical response.

**Extracellular matrix (ECM):** a complex network of proteins and other molecules surrounding cells, providing structural support. In muscle tissue, the ECM transmits forces generated by muscle contraction and maintains tissue integrity.

**Fragile X-associated tremor/ataxia syndrome (FXTAS):** a progressive neurodegenerative disorder that primarily affects older adults, typically males. It is linked to a genetic premutation in the *FMR1* gene on the X chromosome, which is also associated with fragile X syndrome (FXS). Unlike FXS, FXTAS usually begins in adults over the age of 50 and is characterized by progressive cerebellar ataxia (loss of coordination) and tremors, as well as anxiety and dementia. FXTAS is caused by a premutation involving an expansion of 55-200 CGG repeats in the *FMR1* gene. Unlike the full mutation seen in FXS (more than 200 repeats), which typically causes silencing of the gene and loss of the protein FMRP, the premutation increases mRNA levels and is thought to have a toxic effect on neuronal cells, leading to the symptoms of FXTAS.

**Gene modulation:** therapeutic strategies that alter the function of the disease-causing gene for therapeutic benefit. This does not involve replacing or knocking down the gene but modifying its activity or function.

**Glycogen storage disorders:** these disorders are characterized by a defect in the metabolism of glycogen, affecting its synthesis or breakdown.

**Haploinsufficiency:** a disease mechanism where having only one functional copy of a gene is insufficient to maintain normal function.

**Heat shock proteins (HSPs):** a family of proteins that are produced by cells in response to stressful conditions. They function as chaperones, helping to prevent the aggregation of misfolded proteins.

**Hypotrophy:** small muscle fibers resulting from a failure to develop or mature (e.g. nemaline myopathy, centronuclear myopathy and fiber-type disproportion) as opposed to those that are small secondary to atrophy.

**Knockdown:** a technique used to reduce the expression of a gene. It can be achieved through methods like RNA interference (RNAi), which involves the use of short RNA molecules to target and degrade specific mRNA molecules, thereby reducing protein production.

**Kyphosis:** a spinal disorder characterized by an excessive outward curvature of the spine, resulting in a rounded or hunched back.

**Lever arm swing:** often referred to as the power stroke of muscle contraction. Upon the release of ADP and inorganic phosphate, the myosin head undergoes a conformational change, swinging its lever arm. This movement pulls the actin filament along, thus shortening the sarcomere, which is the fundamental action of muscle contraction.

**Linker of nucleoskeleton and cytoskeleton (LINC) complex:** a protein assembly that links the nuclear envelope to the cytoskeleton, facilitating the transmission of mechanical forces and maintaining nuclear positioning within the cell.

**Liquid-liquid phase separation (LLPS):** a process by which a homogeneous solution separates into two distinct liquid phases, allowing for the formation of membrane-less organelles within the cell.

**MicroRNA (miRNA):** small non-coding RNA molecules that function in RNA silencing and post-transcriptional regulation of gene expression.

**Modifier genes:** genes that do not directly cause a disease but can influence the severity, progression or symptoms of a disease when mutated.

**Myofiber:** or muscle fiber, a single muscle cell, which is multinucleated and cylindrical in shape. It is the functional unit of a muscle and is surrounded by a plasma membrane known as the sarcolemma. Each muscle fiber contains many myofibrils that run parallel to its length. Myofibers bundle together to form muscle tissue.

**Myofibril:** the basic rod-like unit of a muscle fiber. Multiple myofibrils are contained within each muscle fiber, and their coordinated contraction leads to the overall contraction of the muscle. Myofibrils are made up of sarcomeres, which are the smallest contractile units within muscle fibers, aligned end-to-end along the length of the myofibril.

**Myofibrillar myopathy:** a group of muscle disorders characterized by the disintegration of myofibrils, the contractile threads of muscle cells, leading to muscle weakness and wasting.

**Myoglobin:** a protein in muscle cells that binds oxygen and facilitates its transport within muscle.

**Nuclear envelope:** the double-membrane structure that encloses the nucleus, separating it from the cytoplasm. It includes nuclear pore complexes for regulated molecular exchange and is connected to the cell's cytoskeleton through the LINC complex.

**Nuclear localization sequence (NLS):** a specific sequence of amino acids in a protein that signals for its transport into the nucleus through nuclear pore complexes.

**Nuclear pore complex (NPC):** a large protein complex that spans the nuclear envelope, controlling the movement of molecules between the nucleus and the cytoplasm.

**Nucleocytoplasmic shuttling:** the process by which proteins and RNAs are transported between the nucleus and the cytoplasm.

**Pes cavus:** an abnormality of the foot, characterized by an abnormally high arch that does not flatten with weight bearing.

**Polyadenylation signal (PAS):** a sequence element in mRNA that signals the addition of a poly(A) tail, which is important for mRNA stability and regulation of translation.

**Prion-like domains (PrLDs):** domains within proteins that have low complexity, with the ability to undergo conformational changes and form reversible aggregates, similar to prions.

**Proteasome:** a protein complex that degrades unneeded or damaged proteins that have been tagged for destruction by ubiquitin. It plays a vital role in maintaining the cell's overall protein quality.

**Proteostasis:** the regulation of the cellular protein content, including the synthesis, folding, trafficking and degradation of proteins. Proper proteostasis is essential for muscle cell function and health.

**RAN:** an enzyme that is crucial for the transport of proteins across the nuclear envelope, providing directionality for nucleocytoplasmic transport.

**Repeat-associated non-ATG translation:** a process by which repeat expansions within non-coding regions of RNA lead to the production of potentially toxic proteins without a traditional starting ATG codon.

**RNA-binding proteins (RBPs):** proteins with RNA recognition motifs that play roles in RNA processing, including transcription, splicing, transport and degradation.

**RNA foci:** aggregates of mutant RNA that accumulate and can sequester RNA-binding proteins, leading to disruptions in RNA processing.

**RNA interference (RNAi):** a biological process by which RNA molecules inhibit gene expression by causing the degradation of specific mRNA molecules.

**RNA splicing:** the process by which introns are removed from pre-mRNA and exons are joined together to form mature mRNA.

**Sarcomere:** the basic unit of striated muscle tissue responsible for muscle contraction. It is a highly ordered assembly of actin and myosin filaments bounded by Z-discs.

**Scapuloperoneal:** a specific distribution of muscle weakness that affects both the shoulder (scapular) and lower leg (peroneal) regions. Weakness of scapula-stabilizing muscles can lead to difficulties in lifting the arms and can cause the shoulder blades to wing out abnormally from the back. Peroneal weakness involves muscles responsible for lifting the foot at the ankle (dorsiflexion) and can lead to foot drop. Scapuloperoneal patterns of weakness can be seen in several genetic muscle diseases such as Emery-Dreifuss muscular dystrophy and others.

**Store-operated calcium entry (SOCE):** a cellular mechanism by which the depletion of calcium stores in the sarcoplasmic reticulum triggers an influx of extracellular calcium through plasma membrane channels. This process is crucial for maintaining calcium levels necessary for various cellular functions, including muscle contraction.

**Stress granules:** cytoplasmic aggregates of proteins and RNAs that form in response to cellular stress and are involved in the regulation of mRNA stability and translation.

**Toxic/gain of function:** mutations that endow a protein with a new, often detrimental, function that contributes to disease pathology. This can involve increased protein activity, abnormal stability or the acquisition of toxic properties unrelated to the protein's normal function.

**Triad complex:** a structure composed of a T-tubule flanked by two terminal cisternae of the sarcoplasmic reticulum. It plays a crucial role in coupling depolarization of the muscle cell membrane to the release of calcium from the sarcoplasmic reticulum.

**Type 1 fiber predominance:** a histopathological abnormality of muscle where a majority of muscle fibers are type 1.

**Ubiquitin:** a small regulatory protein that is attached to substrates and marks them for degradation by the proteasome.

**Ubiquitin ligases (E3):** enzymes that recognize specific protein substrates and catalyze the attachment of ubiquitin, marking the protein for degradation by the proteasome.

**Fig. 3.**
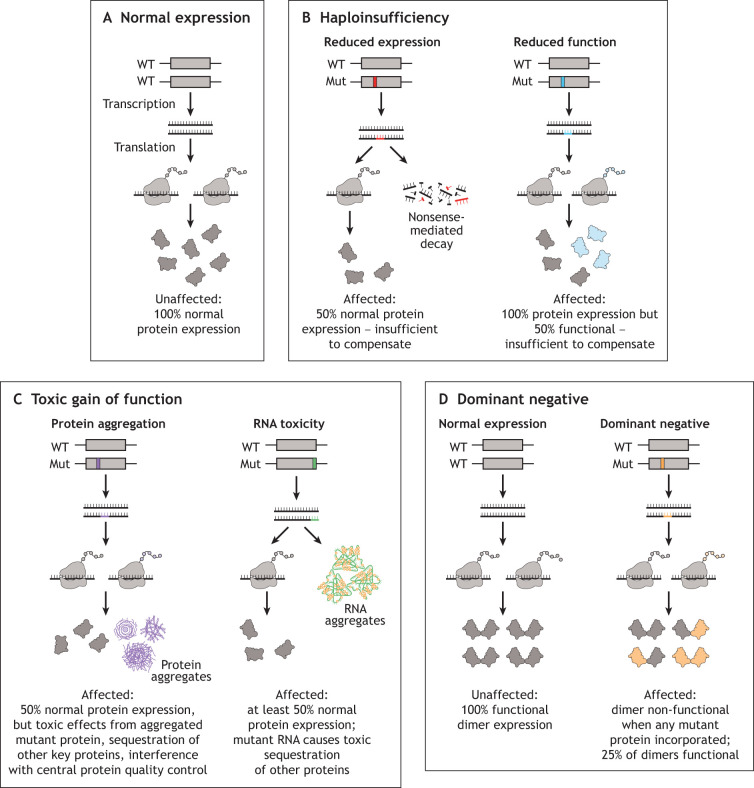
**Dominant disease mechanisms.** (A) Normal expression of protein. (B) Red represents mutations that reduce expression (i.e. nonsense, frameshift, etc.). Blue represents mutations that impact function (i.e. missense, in-frame deletion, etc.). Endogenous functioning proteins are colored gray and non-functioning proteins are colored blue. In disorders of haploinsufficiency, greater than 50% functioning protein is required to prevent disease. (C) Gain-of-function mechanisms refer to those involving increased protein levels (gene duplication or increased stability of mutant protein), hyperactivity of the mutant protein, or from misfolding of the mutant protein (purple) or RNA (green), creating aggregates that are toxic when not degraded. (D) Dominant-negative mechanisms are most easily illustrated in the case of proteins that form dimers or other multimeric structures. Any dimer that contains mutant protein (orange) is rendered non-functional. Assuming that each allele produces an equal amount of protein with equal stability, only 25% of dimers will be functional. Mut, mutant; WT, wild type. Republished with permission. The Creative Commons license does not apply to this content. Use of the material in any format is prohibited without written permission from the publisher, Wolters Kluwer Health, Inc.

Recessively inherited muscle disorders are fairly uniform in their disease mechanisms, as they are either due to mutations causing absence of the protein, or mutations rendering the protein non-functional (Fig. 3A). These mutations are therefore broadly referred to as causing a ‘loss of function’. There are at least three generalized molecular mechanisms that can result in dominantly inherited disorders: haploinsufficiency (Box 1), toxic/gain of function (Box 1) and dominant negative (Box 1) (Fig. 3A-D). These oversimplifications provide a helpful framework for thinking about dominant disease mechanisms (Findlay and Weihl, 2022). It is important to recognize that these broad classifications do not fully convey the mechanistic intricacies of most dominantly inherited conditions, many of which are still under active investigation.

**Fig. 4.**
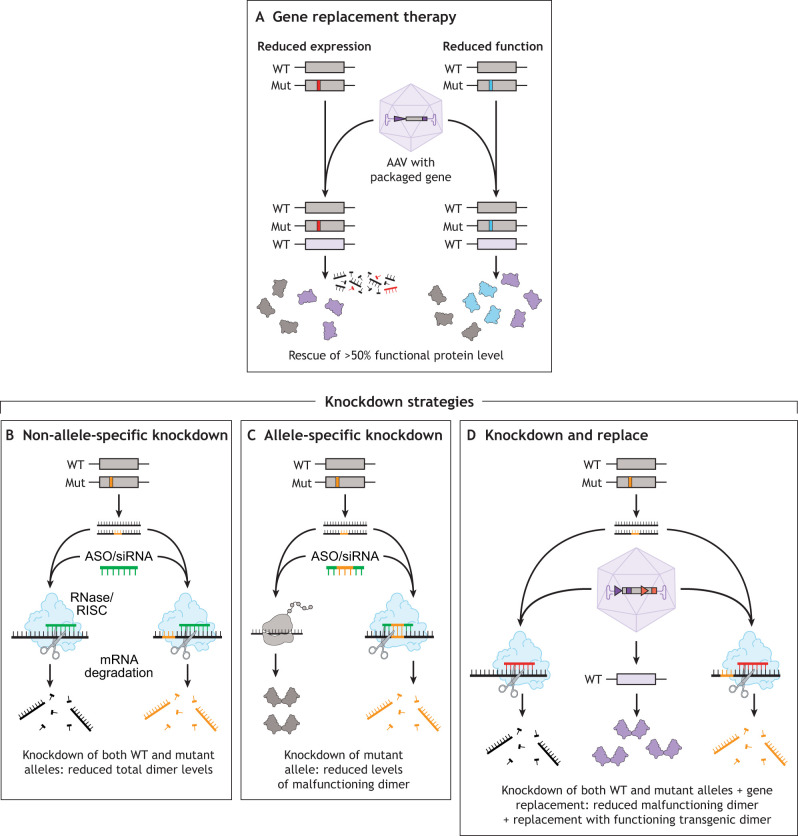
**Gene therapy approaches for dominant disease mechanisms.** (A) Gene replacement therapy for dominant haploinsufficiency disorders due to reduced expression (red mutations) or loss of function (blue mutations). An AAV provides a functional copy of the gene (purple). (B-D) A variety of knockdown strategies may be used to treat dominant-negative or toxic, gain-of-function disorders. (B) Global knockdown of both WT (black) and mutant (black and orange) alleles is an ideal treatment strategy for dominant disorders if absence of the gene is not deleterious. One method for this is via siRNA or certain types of ASOs (green), which cause degradation of the target mRNA via recruitment of RISC or RNase, respectively. (C) Mutant allele-specific knockdown is preferred when knockout is deleterious but haploinsufficiency is tolerated. This can be achieved by designing siRNA (or other antisense knockdown technology) (green and orange), to preferentially bind to the mutant allele (black and orange). (D) In cases in which knockout and haploinsufficiency are both not tolerated, a knockdown-and-replace approach may be required. This can be achieved via viral delivery of RNAi (red) targeting both the WT allele (black) and mutant allele (black and orange) and simultaneous gene replacement using a codon optimized transgene (purple) to avoid knockdown. AAV, adeno-associated virus; ASO, antisense oligonucleotide; Mut, mutant; RISC, RNA-induced silencing complex; siRNA, small interfering RNA; WT, wild type. Republished with permission. The Creative Commons license does not apply to this content. Use of the material in any format is prohibited without written permission from the publisher, Wolters Kluwer Health, Inc.

Gene therapy treatments are not yet clinically available for dominantly inherited myopathies, as there are many key unmet needs and barriers. Many dominantly inherited muscle disorders are rare diseases, making it challenging to attract research funding and expertise. Given their significant genetic heterogeneity, treatments need to be tailored to specific genetic mutations and disease subtypes (Box 2 and Fig. 4). The gene replacement strategies that are commonly employed for recessive, loss-of-function disorders are not readily translatable to most dominant forms of myopathies and muscular dystrophies, owing to the presence of a wild-type (WT) chromosome, which hinders the development of novel treatments (Findlay and Weihl, 2022). Additionally, dominantly inherited disorders have complex, heterogeneous disease mechanisms, each requiring unique therapeutic approaches. Further, the natural history of disease progression for many dominantly inherited myopathies is not well characterized yet (Angelini et al., 2019; Findlay et al., 2022; Ikenaga et al., 2020; Kroon et al., 2021). Defining the natural history of disease progression is crucially important to identify ideal outcome measures for future therapeutic trials.

**Box 2. Gene therapy approaches to address dominant disease mechanisms**

Numerous gene-based therapeutic approaches are available, and the most effective strategy for treating a specific disease is determined by the underlying mechanism of the disorder (Fig. 4) (Findlay and Weihl, 2022). Gene therapies are broadly classified into two main types: those that target the disease-causing gene and those that do not. Treatments that target the gene responsible for the disease may operate at either the DNA or RNA level and are typically categorized into four main types: gene replacement, gene modulation (Box 1), gene correction and gene knockdown.

Gene replacement therapies are effective for treating recessive loss-of-function disorders, such as Duchenne muscular dystrophy (DMD), and dominant haploinsufficiency disorders (Fig. 4A). This therapeutic approach involves supplementing a nonfunctional or absent gene by introducing a functional gene copy, usually delivered to the patient's cells via an adeno-associated virus (AAV). Unlike other gene therapy strategies that require specific mutation types, gene replacement is versatile and can address any mutation that results in a loss of function or expression (Findlay and Weihl, 2022).

Gene modulation involves treatment approaches that modify the disease-causing gene to achieve a therapeutic effect. Unlike gene replacement or knockdown strategies, gene modulation does not introduce a new gene copy or reduce the expression of the gene. These treatments often target specific mutations, which limits their applicability to all patients with a particular disorder. A notable example of gene modulation is the use of antisense oligonucleotides (ASOs) for exon skipping in the treatment of DMD (Findlay and Weihl, 2022).

In contrast, knockdown treatments that reduce levels of the targeted mutant protein are well suited to address dominantly inherited diseases caused by a toxic, gain-of-function, or dominant-negative mechanisms (Findlay and Weihl, 2022). There are a variety of different knockdown approaches (Fig. 4B-D). Which of these is most well suited for a disease depends on how much of the affected protein is required for a cell to function normally. If complete absence of a protein is tolerated, a knockdown approach that targets both copies of a gene (non-allele specific) could be beneficial for a dominantly inherited disease (Fig. 4B). If at least 50% protein levels are required for cellular health, a knockdown treatment that selectively targets the mutant gene (allele specific) can be used. Allele-specific knockdown, although technically challenging, is achievable even when two transcripts vary by just a single base pair. This method involves the precise design of RNA interference or ASO sequences that selectively target and bind to the mutant allele (Fig. 4C). If over 50% protein levels are required for cellular health, then treatment approaches involving complete knockdown of a gene while simultaneously providing a replacement copy of the gene that is resistant to knockdown can be beneficial (Fig. 4D) (Findlay and Weihl, 2022). This strategy uses an AAV to deliver the necessary genetic cargo.

Gene correction techniques such as CRISPR/Cas9, base editing and prime editing offer the possibility of directly repairing mutations responsible for diseases, potentially applicable to any inherited disorder, irrespective of the underlying mechanism.

Therapies that do not target the disease gene may instead focus on modifier genes or homologs of the disease gene. Although modifier genes do not directly cause the disease, they can influence its severity. Targeting these genes aims to adjust or ameliorate the downstream effects commonly associated with myopathies, such as muscle atrophy or fibrosis. Upregulating homologs of disease genes (e.g. *UTRN*) could, in theory, be therapeutic for disorders due to loss-of-function mutations (e.g. *DMD*).

There are several other significant hurdles in the clinical application of gene therapy strategies (Henderson et al., 2024; Lek et al., 2023; McNally, 2024; Shieh et al., 2020; Wilton-Clark and Yokota, 2023). For RNA interference (RNAi; Box 1)- and antisense oligonucleotide (ASO; Box 1)-based therapeutics, many trials have failed owing to poor delivery to skeletal muscle (Hammond et al., 2021). For treatments utilizing adeno-associated virus (AAV; Box 1), one of the primary obstacles is the prevalence of pre-existing anti-AAV antibodies, which could substantially limit the number of patients eligible for clinical trials (Henderson et al., 2024; McNally, 2024). The recent deaths of several patients involved in gene therapy trials highlight the large safety risks associated with the immune response and the importance of understanding a therapy's potential impact on extra-muscular tissues (Lek et al., 2023; Shieh et al., 2020). These incidents emphasize the importance of balancing the potential benefits of gene therapy with these safety risks. There is also a pressing concern regarding how the healthcare system will manage the substantial costs associated with gene therapies, especially considering the vast population of patients with rare diseases who could potentially benefit from such treatments (Henderson et al., 2024). Despite these hurdles, several studies are currently in preclinical development for disorders such as facioscapulohumeral muscular dystrophy, myotonic dystrophy and centronuclear myopathy due to DNM2 mutations. Knockdown (Box 1) strategies are currently a reality for other dominantly inherited neuromuscular disorders, such as transthyretin-related amyloidosis and amyotrophic lateral sclerosis, owing to SOD1 mutations (Miller et al., 2022).

This Review will focus on rare dominantly inherited skeletal muscle disorders and discuss the key mechanistic themes as they pertain to muscle biology. We will also examine their genetic causes, clinical features, our current understanding of the disease mechanisms (Table 1), and any treatment approaches designed to address the underlying mechanistic defect (Table 2). There are several categories of muscle disease that have been reviewed elsewhere and will not be covered here, such as acquired disorders of muscle (sarcopenia, cachexia, inflammatory myopathies, etc.), muscle channelopathies (periodic paralyses, non-dystrophic myotonias, etc.) (Trivedi, 2022), mitochondrial myopathies (nuclear and mitochondrially encoded) (Cohen, 2019), dominant myasthenic syndromes (Finsterer, 2019) and dominant cardiomyopathies without skeletal muscle involvement (Bang et al., 2022).

**Table 1.**
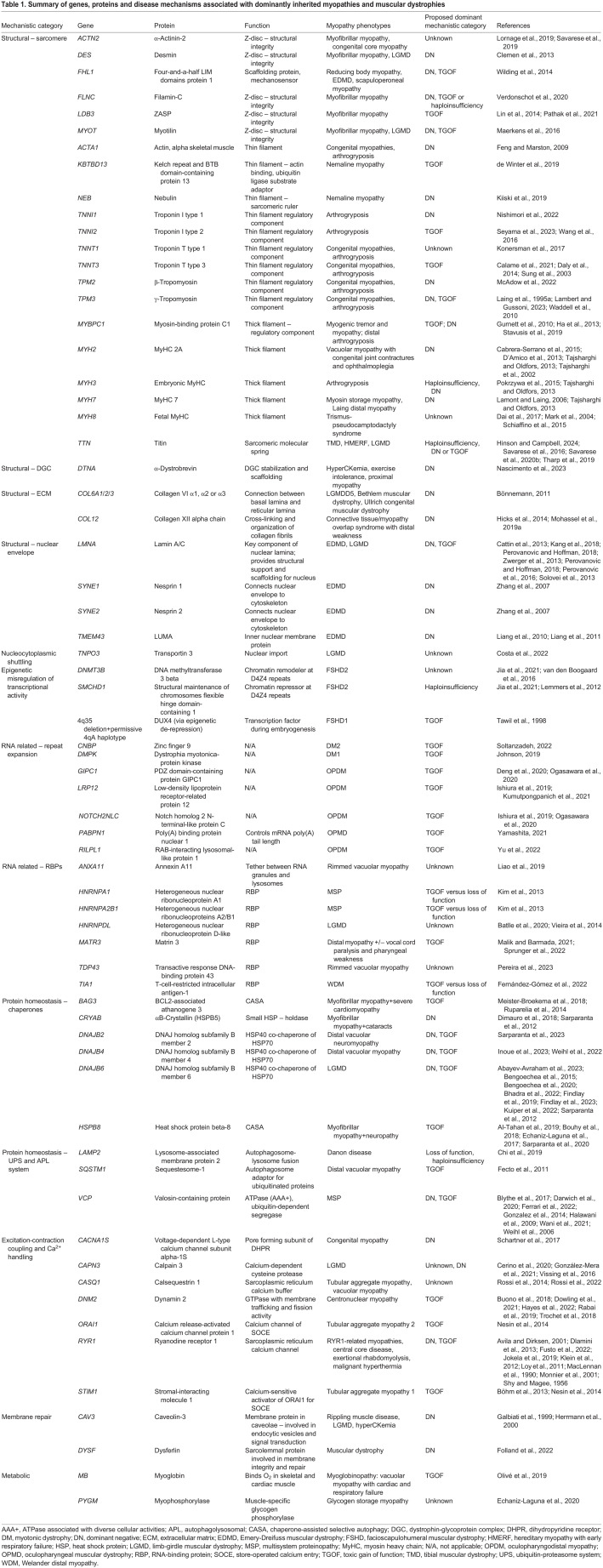
Summary of genes, proteins and disease mechanisms associated with dominantly inherited myopathies and muscular dystrophies

**Table 2.**
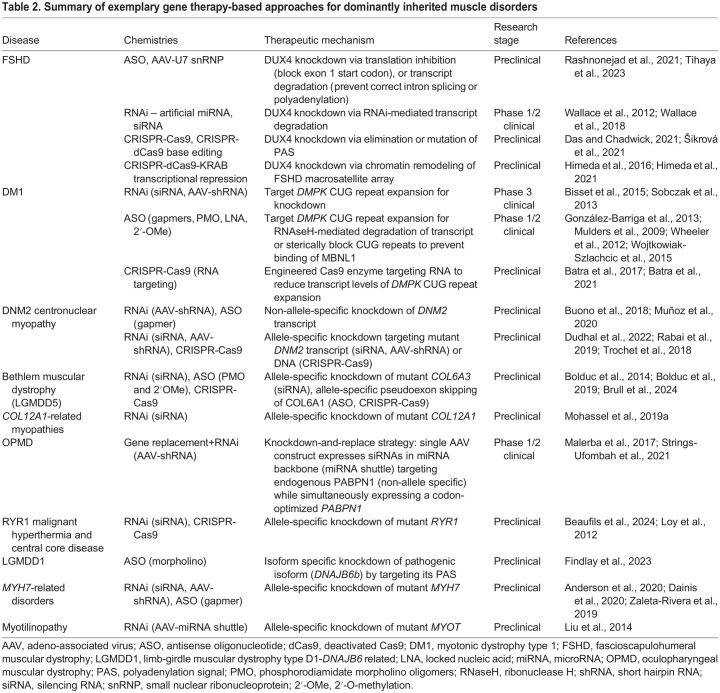
Summary of exemplary gene therapy-based approaches for dominantly inherited muscle disorders

## Disease mechanism categories as they pertain to muscle biology

Similar to recessively inherited disorders, many dominantly inherited muscle disorders are caused by mutations in genes encoding muscle proteins with a wide variety of functions, such as maintaining myofibrillar structure, protein homeostasis, excitation-contraction coupling (Box 1) and Ca^2+^ handling, membrane repair and metabolism. However, some dominantly inherited myopathies are caused by unstable repeat expansion mutations, leading to RNA-mediated toxicity (e.g. myotonic dystrophy), or mutations that lead to epigenetic deregulation of genes that are normally silenced (e.g. FSHD). The following section will summarize what is known about the genetic etiology, clinical features, pathomechanisms, and any current treatment strategies for dominant myopathies and muscular dystrophies (Table 1).

## Skeletal muscle cellular structural components

The skeletal muscle cytoskeleton (Box 1) enables the precise execution of muscle contractions. Each myofiber (Box 1) houses various cytoskeletal structures. The sarcomere (Box 1) is formed by a multitude of both structural and regulatory proteins. Intermediate filaments connect the sarcomere with organelles like mitochondria and nuclei, providing a structural framework and facilitating mechanotransduction (Henderson et al., 2017). The linker of nucleoskeleton and cytoskeleton (LINC) complex (Box 1), establishes a physical connection between the interior of the nucleus, the nuclear envelope (Box 1) and the cytoskeleton. The sarcomeric Z-disc and M-band are anchored to the sarcolemma, the enclosing membrane of the myofiber, by the costamere complex. The costamere is composed of two major protein complexes: the dystrophin-glycoprotein complex (DGC; Box 1) and the integrin-vinculin-talin complex. Analogous to the LINC complex's role spanning the nuclear membrane, the sarcolemmal DGC and integrin-vinculin-talin complex effectively connect all internal myofibrillar cytoskeletal structures to the extracellular matrix (ECM; Box 1) (Henderson et al., 2017; Wilson et al., 2022). The coordinated action of all these key structural components (nuclear envelope, sarcomere, costameric complexes and ECM) is essential for proper muscle function, and any disturbances in their integrity can lead to skeletal muscle disorders.

### Structural – sarcomere

The sarcomere is the fundamental contractile unit within striated muscles. Its outer limits are marked by Z-discs. Flanking each Z-disc are I-bands, containing titin as well as thin filament proteins such as actin. The A-band separates I-bands, and encompasses the full length of myosin-filled thick filaments, and the M-band is located at its midpoint (Henderson et al., 2017). Muscle contraction is driven by the myosin motors binding actin thin filaments and drawing the Z-discs towards the M-band. The sarcomere is a dynamic entity; although it was once thought to be a rigid framework for regulatory and structural proteins, it is currently understood to be capable of considerable and swift protein turnover, adapting to changes in muscle stress and repairing itself post-injury. Disruption to several components of the sarcomere have been associated with skeletal muscle disorders.

#### Z-discs

Z-discs connect the pointed ends of adjacent sarcomeres' actin thin filaments via α-actinin. They provide attachment points for the N-terminal ends of titin and nebulin/nebulette filament systems (Henderson et al., 2017). The Z-disc and its complex network of proteins are also actively involved in signal transduction and protein turnover. Dominantly inherited mutations in Z-disc proteins, such as those discussed below, have been implicated in a variety of cardiomyopathies, muscular dystrophies and distal myopathies. Histopathologically, many of these disorders have overlapping features, including myofibrillar disorganization, protein aggregation and rimmed vacuoles.

Intermediate filaments play a crucial role in maintaining the structural integrity of muscle fibers. The structure of intermediate filament proteins includes a head at the N-terminal, a tail at the C-terminal, and a central rod-like domain that twists into a coil, forming a dimer. These dimers come together to form either homo- or hetero-oligomers (Henderson et al., 2017). Desmin is the predominant intermediate filament protein specific to muscle, constituting 0.35% of the total protein in skeletal muscle and 2% in cardiac muscle. Desmin localizes to the periphery of Z-discs, forming a network with itself and other proteins like vimentin, nestin, synemin or paranemin (Henderson et al., 2017). This network connects Z-discs of neighboring myofibrils (Box 1), as well as linking them to membrane adhesion complexes, costameres, the sarcolemma, mitochondria and nuclei. Desmin is also present at the M-band, where it helps in aligning myofibrils laterally (Henderson et al., 2017). Despite desmin being one of the initial muscle-specific proteins expressed during myofibrillogenesis, it is not essential for muscle development *in vivo*. Desmin knockout mice exhibit normal muscle differentiation, cell fusion and maturation. Postnatally, however, these mice experience issues such as misaligned myofibrils, disrupted costameres, abnormal nuclear shape and positioning, mitochondrial irregularities and reduced force generation (Capetanaki et al., 1997; Milner et al., 1996). Desmin's critical role in maintaining cytoskeletal integrity is well recognized given its involvement in both skeletal and cardiac diseases, termed desminopathies. These disorders are characterized by disorganization of desmin filament networks, with vacuoles and insoluble subsarcolemmal aggregates of desmin (Goldfarb et al., 2008). Desminopathies can present in various forms, with some cases exhibiting a progressive adult-onset distal predominant myopathy, sometimes accompanied by cardiac symptoms (Dalakas et al., 2000). In other instances, cardiomyopathy, congestive heart failure, heart block and arrhythmias are the primary or sole symptoms. Although most cases of desmin myopathy are inherited in an autosomal-dominant pattern, there are rare instances of autosomal-recessive inheritance (Dalakas et al., 2000; Goldfarb et al., 2008). Most desmin mutations affect the coil 2B domain, which is critical for intermediate filament assembly. These desmin mutants cannot form an intermediate filament network and instead aggregate and collapse the pre-existing intermediate filament network (Bär et al., 2005). Mice lacking desmin are free of desmin aggregates, but develop cardiomyopathy and skeletal myopathy, suggesting that the primary mechanism for dominantly inherited mutations is likely to be a dominant-negative effect on desmin and its interacting molecules, rather than a toxic function of desmin aggregates (Clemen et al., 2013).

The filamin protein family includes filamin-A (α-isoform), filamin-B (β-isoform) and the muscle-specific filamin-C (γ-isoform). These proteins organize actin filaments via their N-terminal actin-binding domain. They have a central rod domain, and a C-terminal domain that facilitates dimerization (Henderson et al., 2017). *FLNC* encodes filamin-C, which features a distinct 81-amino acid insertion in its rod domain, aiding in its localization to the Z-disc, where it connects to costameres, participates in integrin-mediated signal transduction processes, and interacts with γ- and δ-sarcoglycans from the DGC and Z-disc proteins such as myotilin and FATZ. The activity of filamin-C is modulated by calpains-1 and -3, calcium-dependent enzymes that cleave filamin-C, affecting its interaction with sarcoglycans (Henderson et al., 2017). Dominantly inherited mutations in *FLNC* cause a variety of myopathic phenotypes, including an adult-onset, slowly progressive distal myopathy, typically presenting with weakness of grip then ankle plantar flexion (Duff et al., 2011; Verdonschot et al., 2020). Mutations also cause a variety of cardiomyopathies. Certain mutations involving the actin-binding domain may cause disease through a gain of function by enhancing actin-binding activity (Verdonschot et al., 2020). Other mutations impact its dimerization domain, preventing it from forming homodimers, leading to its aggregation in skeletal muscle and likely causing disease via a dominant-negative effect. There are also truncating variants associated with cardiomyopathy that may cause disease via haploinsufficiency (Verdonschot et al., 2020).

The *MYOT* gene produces myotilin, a crucial component of the Z-disc in muscle fibers. Patients typically develop distal weakness involving ankle dorsiflexion and plantarflexion after the age of 50 (Hauser et al., 2000). Some patients develop an LGMD, but distal myopathy remains the primary phenotype. Histopathologically, it is characterized by myofibrillar myopathy (Box 1), with vacuoles, aggregates containing myotilin, and myofibrillar disorganization. Although absence of myotilin does not appear to disrupt muscle structure or function, dominant mutations in myotilin have been suggested to cause disease via either a dominant-negative or toxic gain-of-function mechanism (Maerkens et al., 2016; Moza et al., 2007). Interestingly, microRNA (miRNA; Box 1)-mediated knockdown of mutant myotilin in a mouse model that overexpresses mutant human myotilin resulted in both functional and myopathological improvements (Table 2) (Liu et al., 2014).

Lim domain-binding 3 (*LDB3*) encodes Z-band alternatively spliced PDZ-motif-containing protein (ZASP), a PDZ-LIM family protein. PDZ and LIM domains act as scaffolds for Z-disc protein-protein interactions (Henderson et al., 2017). Similar to myotilinopathy, dominant mutations in *LDB3* cause a late-onset distal myopathy (Markesbery-Griggs myopathy, zaspopathy), starting primarily with ankle dorsiflexion weakness and later spreading to proximal muscles (Selcen and Engel, 2005). Cardiomyopathy may develop much later, and facial and respiratory muscles are generally unaffected. Muscle biopsies demonstrate myofibrillar disorganization with vacuoles (Savarese et al., 2020a; Selcen and Engel, 2005). Dominant mutations can also lead to cardiomyopathies. A toxic gain-of-function mechanism has been suggested, but haploinsufficiency is unlikely as mutations do not impact ZASP expression levels and haploinsufficient mice have normal muscle function (Lin et al., 2014; Pathak et al., 2021).

Four-and-a-half LIM (FHL) proteins 1, 2 and 3, are largely expressed in cardiac and skeletal muscle. FHL1 localizes to the M-band, I-band, Z-disc, nucleus and cytoplasm (McGrath et al., 2006). It serves as a mechanosensor by interacting with titin at its N2B spring region and activating downstream NFATC1-mediated hypertrophy signaling pathways in response to muscle strain (Henderson et al., 2017). Hypertrophy of striated muscle is blunted in *Fhl1*-deficient mice (Sheikh et al., 2008). Mutations in *FHL1* cause several forms of X-linked dominant disorders, including Emery-Dreifuss muscular dystrophy (EDMD), X-linked scapuloperoneal myopathy, reducing body myopathies, rigid spine syndromes and sometimes hypertrophic cardiomyopathy (Quinzii et al., 2008). Reducing body myopathies can manifest early in childhood with severe proximal predominant weakness, respiratory involvement, contractures, dysphagia (difficulty swallowing; Box 1) and death sometimes before the age of 10 (Quinzii et al., 2008). Other mutations cause a scapuloperoneal pattern of weakness starting as late as age 40, with associated weakness of paraspinal muscles, spine rigidity, scoliosis, respiratory failure and dilated cardiomyopathy. Females are commonly symptomatic but have less severe disease than males. Muscle biopsy demonstrates myopathic changes, rimmed vacuoles and reducing bodies, which are cytoplasmic inclusions containing desmin, ubiquitin (Box 1), FHL1 and GRP78 that reduce nitro-blue tetrazolium (NBT), resulting in them staining dark with menadione-NBT (Schessl et al., 2008). Mutations in distal exons that encode the third and fourth LIM domains, as well as frameshift mutations leading to reduced FHL1 expression, are associated with non-reducing body disorders such as hypertrophic cardiomyopathy and EDMD. Mutations associated with reducing body myopathies are thought to cause disease either through a dominant-negative or toxic gain-of-function mechanism that can be rescued by NFATC1 expression (Wilding et al., 2014).

α-Actinin (ACTN) isoforms are members of the spectrin superfamily that were originally described to function by cross-linking actin filaments. There are four overlapping genes (*ACTN1-4*). *ACTN2* and *ACTN3* encode skeletal muscle isoforms, and *ACTN2* is also found in cardiac muscle (Henderson et al., 2017). ACTN2 is situated in the Z-disc and functions as a dimer, where it binds actin and titin filaments and provides a structural framework for other Z-disc proteins. It senses mechanical stress and facilitates various signaling pathways (Henderson et al., 2017). Mutations in the *ACTN2* gene were previously linked to hypertrophic and dilated cardiomyopathies. More recently, dominantly inherited mutations were found to cause an adult-onset distal myopathy with vacuoles, inclusions and myofibrillar changes, as well as a childhood-onset myopathy with respiratory involvement and muscle fibers containing small-structured cores and jagged Z-lines (Lornage et al., 2019; Savarese et al., 2019). The exact underlying mechanism remains unclear, but evidence does not support haploinsufficiency (Lornage et al., 2019; Savarese et al., 2019).

#### Thin filament

The thin filament is primarily composed of skeletal muscle actin (encoded by *ACTA1*), which accounts for 20% of the mass of skeletal muscle. Individual actin molecules (G-actin) interact to form highly dynamic filamentous polymers (F-actin) (Henderson et al., 2017). F-actin's barbed end inserts into the Z-disc and its pointed end extends into the M-band. Thin filaments are also composed of a group of proteins that regulate its length and provide dynamic interactions with the thick filament. These include nebulin, tropomyosin and the troponin complex subunits: isoforms of troponin T (TNNT; bind tropomyosin), troponin I (TNNI; bind actin) and troponin C (TNNC; bind calcium ions) (Henderson et al., 2017). Mutations in many thin filament proteins, as described below, cause nemaline myopathy, a form of congenital myopathy, typically characterized by non-progressive diffuse weakness, reduced muscle bulk, and a predilection for involvement of the bulbar musculature (Box 1) and axial musculature (Box 1) (Amburgey et al., 2021; Dowling et al., 2021). Muscle biopsies also typically show myofiber hypotrophy (Box 1), type 1 fiber predominance (Box 1), myofibrillar disorganization and nemaline rods, which consist of aggregated actin and Z-disc material (Amburgey et al., 2021; Dowling et al., 2021).

Mutations in *ACTA1* are the most common cause of dominant congenital myopathies (Agrawal et al., 2004; Hutchinson et al., 2006). They cause a range of myopathological changes, with nemaline myopathy being the most common. Most disease-associated variants are missense changes that impair actin filament polymerization and stability, causing reduced maximal force generation, resulting from reduced thin filament length or function (Dowling et al., 2021; de Winter et al., 2016). Recessively inherited loss-of-function or -expression mutations also cause disease. In general, most *ACTA1* mutations are thought to cause pathology via a dominant-negative mechanism (Feng and Marston, 2009).

*TPM3* (Laing et al., 1992, 1995a), *TPM2* (Mokbel et al., 2013; Tajsharghi et al., 2007), *TNNT1* (Konersman et al., 2017), *TNNT3* (Gurnett et al., 2009; Sung et al., 2003), *TNNI1* (Nishimori et al., 2022) and *TNNI2* (Seyama et al., 2023; Wang et al., 2016) are all regulatory components of the thin filament. Dominant mutations are associated with a variety of congenital myopathies (nemaline myopathy, congenital fiber type disproportion, cap myopathy) or distal arthrogryposis, a syndrome of congenital joint contractures that likely results from weakness and lack of limb movement during embryogenesis. *TPM2* encodes β-tropomyosin, which is primarily expressed in both slow and fast-twitch muscle fibers, whereas *TPM3* encodes γ-tropomyosin, which is expressed solely in slow-twitch muscle fibers (Lambert and Gussoni, 2023). Most disease-causing mutations in *TPM2* and *TPM3* cause disease by disrupting tropomyosin-actin interactions. This may occur via a gain of function, in the case of *TPM2*, and either a dominant negative or gain of function for *TPM3* (Laing et al., 1992, 1995a; Lambert and Gussoni, 2023; McAdow et al., 2022; Waddell et al., 2010).

Pathogenic mutations in *TNNI2*, which encodes troponin I2, cause gain-of-function changes with increased ATPase activity in actin-activated myosin ATPase assays, leading to contractures and limb deformities via increased tension in developing muscles (Seyama et al., 2023; Wang et al., 2016).

*NEB* encodes nebulin, a large protein that interacts with many other sarcomeric proteins, such as titin, α-actinin and desmin at its C-terminus in the Z-disc. Its N-terminus interacts with tropomodulin at the end of the thin filament. Its middle is composed of 22 seven-module super-repeats that bind actin, tropomyosin and others (Henderson et al., 2017). Nebulin acts as a molecular ruler and actin stabilizer. Altering nebulin size causes corresponding changes in thin filament length (Kiss et al., 2020; Witt et al., 2006). Loss of nebulin causes reduced thin filament length, which decreases the overlap between thin and thick filaments, impairing force generation, leading to weakness (Kiss et al., 2020; de Winter et al., 2016; Witt et al., 2006). Nemaline myopathy is most commonly caused by recessive *NEB* mutations (Laitila and Wallgren-Pettersson, 2021; Pelin et al., 1999). There is, however, a dominantly inherited deletion involving a large portion of *NEB* that causes a distal predominant nemaline/cap myopathy. This mutation produces a smaller nebulin protein, which may cause disease via a dominant-negative mechanism (Kiiski et al., 2019).

Kelch domain-containing proteins facilitate substrate specificity for cullin-3, an E3 ubiquitin ligase (Box 1). Recessive mutations in Kelch proteins (KLHL40, KLHL41) are thought to cause nemaline myopathies by impairing efficient polyubiquitination of their thin filament substrates (NEB, LMOD3, NRAP), leading to their reduced turnover. Dominant point mutations in *KBTBD13* cause nemaline myopathy 6 (NEM6), which is clinically characterized by weakness and slow muscle relaxation (Sambuughin et al., 2010). KBTBD13 is a muscle-specific protein with an N-terminal BTB domain that interacts with cullin-3 and a C-terminal Kelch-repeat domain that acts as an adaptor for substrates to be ubiquitinated (Gupta and Beggs, 2014; Sambuughin et al., 2012). KBTBD13 also has functions outside of the ubiquitin-proteasome (Box 1) system. Specifically, it binds to thin filament actin and modulates the relaxation kinetics of muscle (de Winter et al., 2019). This is fitting with other nemaline myopathies that result from pathology of the thin filament. Most disease-causing mutations affect the C-terminal Kelch-repeat domain, which result in stiffer and more tightly packed actin filaments, impairing muscle-relaxation kinetics and reducing contractile force (de Winter et al., 2019). KBTBD13 knockout mice have few differences from WT and do not recreate the disease phenotype seen in knock-in KBTBD13 mice with a heterozygous point mutation. The authors suggested a gain-of-function mechanism by which the actin thin filament's biophysical properties are altered via direct binding with the C-terminal Kelch-repeat domain of mutant *KBTBD13* (de Winter et al., 2019). However, the authors also noted that disease-causing mutations exist in the cullin-3-interacting BTB domain, suggesting that altered ubiquitination of actin thin filaments might contribute to disease pathogenesis in certain instances (de Winter et al., 2019).

#### Thick filament

The thick filament is composed of myosin hexamers: two myosin heavy chains (MyHCs), two essential light chains and two regulatory light chains. Myosin slides over the thin filament in an ATP-dependent manner, generating muscle force. The globular head domain of MyHCs interacts directly with actin and has ATPase activity. The rod domain is important for homodimerization and other protein interactions (Henderson et al., 2017).

*MYH2* encodes MyHC 2A, which is found exclusively in type 2A fibers. Dominant mutations cause early-onset distal contractures with later-onset proximal predominant weakness, ophthalmoplegia and dystrophic muscle pathology, along with rimmed vacuoles, protein aggregates and small, but present, type 2A fibers (D'Amico et al., 2013; Findlay et al., 2018; Tajsharghi et al., 2002). Recessive *MYH2* mutations cause ophthalmoplegia, facial weakness and childhood-onset diffuse non-progressive weakness. Contractures are typically absent, and myopathology demonstrates type 1 predominance with reduced or complete absence of type 2A fibers (Findlay et al., 2018). Most dominant mutations involve the coiled-coil rod domain and exert a dominant-negative effect by altering assembly of myosin homodimers (Cabrera-Serrano et al., 2015; D'Amico et al., 2013; Findlay et al., 2018). Another dominant mutation impacts the SH1 helix, critical for the lever arm swing (Box 1) and is also thought to cause a dominant-negative effect via perturbation of conformational changes during ATP hydrolysis (Tajsharghi and Oldfors, 2013; Tajsharghi et al., 2002).

*MYH7* encodes the slow-twitch MyHC 7 isoform, which is expressed in type 1 skeletal muscle fibers and cardiac muscle. Dominant *MYH7* mutations can cause myopathies with several weakness patterns (scapuloperoneal, distal, limb-girdle), as well as isolated cardiomyopathies. Myosin storage myopathy is a protein aggregate myopathy associated with dominant-negative mutations in the distal tail of MyHC 7, leading to impaired assembly of MyHC 7 homodimers and its accumulation between myofibrils and beneath the sarcolemma (Tajsharghi et al., 2003). Disease onset is often during childhood but may occur in adulthood. Weakness is typically slowly progressive in a scapuloperoneal or limb-girdle pattern (Tajsharghi and Oldfors, 2013). Laing distal myopathy (*MYH7*-associated distal myopathy) is similarly caused by dominantly inherited mutations in *MYH7* (Laing et al., 1995b; Meredith et al., 2004). Symptoms often begin in childhood, but onset ranges from infancy to 50 years of age. Weakness starts in the ankle dorsiflexors and progresses to involve finger extensors and neck flexors. Later, facial and proximal limb muscles become weak, although most patients remain ambulant. Unlike myosin storage myopathy, muscle histopathology in Laing distal myopathy patients is variable and may include dystrophic changes, type 1 fiber predominance, minicores, necrosis, mitochondrial abnormalities, rimmed vacuoles and filamentous inclusions. It is not understood why myosin storage myopathy and Laing distal myopathy have varying muscle histopathology. Although myosin storage myopathy mutations typically involve exons 37-40 and impact MyHC filament assembly, mutations associated with Laing distal myopathy are often located in exons 32-36, which encode the mid rod domain that is not critical for filament assembly. Still, these mutations are also thought to have a dominant-negative effect, but likely via a different impact on thick filament structure and function (Lamont and Laing, 2006; Tajsharghi and Oldfors, 2013). A variety of allele-specific (Box 1) knockdown approaches targeting mutant *MYH7* using ASOs, small interfering RNAs (siRNAs) or short hairpin RNA (shRNA) delivered via AAV, have demonstrated improvements in models of *MYH7*-related disorders (Table 2) (Anderson et al., 2020; Dainis et al., 2020; Zaleta-Rivera et al., 2019).

*MYBCP1* encodes slow myosin binding protein-C, which plays a role in stabilizing thick filaments and regulating cross-bridge cycling. Dominant gain-of-function mutations that enhance myosin binding and interfere with cross-bridge kinetics cause a mild myopathy with a tremor of myogenic origin (Stavusis et al., 2019). Dominant-negative mutations cause distal arthrogryposis type 1, characterized by congenital contractures of the hands and feet (Gurnett et al., 2010; Ha et al., 2013).

#### Titin filament

The *TTN* gene encodes titin, a protein that spans half of the sarcomere, acts as a molecular spring and creates the passive mechanical properties of myofibers. Titin is involved in numerous protein interactions, including with calpain-3, and is capable of producing many isoforms through alternative splicing, which are specific to developmental stages or tissues (Henderson et al., 2017; Savarese et al., 2018, 2020a). *TTN* mutations, collectively known as ‘titinopathies’, lead to a variety of conditions affecting skeletal muscle, the heart or both (Hackman et al., 2002). Dominant titinopathies include tibial muscular dystrophy (TMD) and hereditary myopathy with early respiratory failure (HMERF) (Savarese et al., 2016, 2020a). TMD typically begins after age 35 and causes slowly progressive asymmetric ankle dorsiflexion weakness (Hackman et al., 2002). Muscle imaging often reveals fatty degeneration in the anterior tibial muscles, eventually affecting other muscles such as the hamstrings and medial gastrocnemius. Myopathology shows myopathic changes with rimmed vacuoles and protein aggregates reactive for markers of altered protein homeostasis (ubiquitin, P62, LC3). *TTN* mutations also cause several recessive forms of congenital myopathy (centronuclear myopathy, myopathy with cores) and LGMD (Savarese et al., 2020a,b). The clinical spectrum of recessive titinopathies is broad, ranging from early onset with delayed motor milestones and impaired ambulation, to later-onset forms with moderate diffuse weakness (Savarese et al., 2020a,b). Depending on their impact on titin structure and function, dominant *TTN* mutations have been reported to cause disease via haploinsufficiency, dominant-negative or toxic mechanisms (Hinson and Campbell, 2024; Savarese et al., 2016, 2020b; Tharp et al., 2019). At present, there are no therapies for the titinopathies.

### Structural – DGC

The DGC is a multiprotein sarcolemmal complex that links the intracellular cytoskeleton of muscle fibers with the ECM, transmitting muscle contraction forces to the tendon, thereby protecting the sarcolemma from contraction-induced damage (Henderson et al., 2017; Wilson et al., 2022). Although mutations in DGC-linked genes are a common cause of many different hereditary muscle disorders (dystrophinopathies, sarcoglycanopathies, dystroglycanopathies, etc.), nearly all of them are recessively inherited (autosomal or X linked) and due to a loss of function.

*DTNA* encodes α-dystrobrevin, a scaffold protein that stabilizes the DGC by binding dystrophin and α-syntrophin. Mutations that alter α-dystrobrevin's ability to interact with α-syntrophin or localize to the DGC are thought to have a dominant-negative effect, destabilizing other components of the DGC (Nascimento et al., 2023). These mutations have been associated with several clinical manifestations, including hyperCKemia, myalgias, exercise intolerance and childhood-onset proximal muscle weakness (Nascimento et al., 2023).

### Structural – ECM

Muscle fibers are encased in a connective tissue known as the basal lamina, composed mainly of laminin α2, collagen IV, heparan sulfate proteoglycan, fibronectin and perlecan. The sarcolemmal DGC and integrin-vinculin-talin complex bind to laminin α2 within the basal lamina, which then connects to the reticular lamina (collagen I and III) via collagen VI. The basal and reticular laminae work in tandem to maintain the structural integrity of the sarcolemma and facilitate the transfer of contractile forces (Holmberg and Durbeej, 2013; Zhang et al., 2021). The ECM is a complex network of proteins that are essential across numerous tissues (Chakravarti et al., 2022; Lamandé and Bateman, 2020; Mavropalias et al., 2023). Specific mutations in ECM proteins that are abundant in skeletal muscle, such as COL6A, COL12A and LAMA2, can lead to inherited muscular disorders (Ahmad et al., 2020; Chakravarti et al., 2022; Lamandé and Bateman, 2020). Dominantly inherited myopathies associated with ECM proteins are far less numerous than those involving intramyofibrillar proteins (Chakravarti et al., 2022; Lamandé and Bateman, 2020). This difference may arise because the widespread expression of ECM proteins often manifests in phenotypes that affect multiple organ systems, especially skin, blood vessels, bone and cartilage, in which expression is particularly high (Ahmad et al., 2020; Chakravarti et al., 2022; Lamandé and Bateman, 2020). Consequently, muscular symptoms in multisystem ECM disorders may be clinically under-recognized or overshadowed by the more pronounced effects on other organs (Ahmad et al., 2020; Chakravarti et al., 2022; Lamandé and Bateman, 2020).

Bethlem muscular dystrophy (LGMDD5) is caused by dominant-negative mutations in the α1, α2 or α3 subunits of collagen type VI (*COL6A1*, *COL6A2*, *COL6A3*) (Bönnemann, 2011; Jöbsis et al., 1996). Mutations disrupt the multimerization of collagen subunits into collagen VI microfibrils. In addition to slowly progressive proximal predominant weakness, patients also commonly develop contractures and skin changes such as keratosis pilaris. Myopathology shows typical myopathic and dystrophic changes with increasing amounts of connective tissue and fat replacing muscle as the disease progresses. Additionally, collagen VI staining will either be reduced or mislocalized when co-stained with a basement membrane marker (laminin, collagen IV, etc.). Ullrich's congenital muscular dystrophy (UCMD) is an allelic disorder characterized by severe congenital-onset weakness, proximal joint contractures, hyperlaxity of distal joints, severe respiratory insufficiency, and skin abnormalities such as keratosis pilaris and atrophic scars (Bönnemann, 2011). UCMD is typically caused by dominant-negative *de novo* mutations in *COL6A1-3* and less commonly via recessive loss-of-function mutations. Selective knockdown of just the mutant allele (Box 1) is a potential treatment strategy for dominant collagen VI-related dystrophies and has, in fact, shown some efficacy in preclinical studies (Table 2) (Bolduc et al., 2014; Brull et al., 2024).

*COL12A1* encodes the alpha chain of collagen XII, which plays a role in cross-linking collagen fibrils in the ECM of dense connective tissues. Dominant-negative mutations that impact collagen XII fibrillar assembly can cause a congenital syndrome of mild hypotonia, proximal weakness and joint hyperlaxity, as well as a connective tissue/myopathy overlap syndrome with distal predominant weakness (Hicks et al., 2014; Mohassel et al., 2019a). Allele-specific knockdown of mutant *COL12A1* using siRNA has demonstrated efficacy in a human *in vitro* model (Table 2) (Mohassel et al., 2019b).

### Structural – nuclear envelope

The nuclear envelope of myonuclei, composed of two closely juxtaposed lipid bilayers, is essential in maintaining the structural integrity and proper functioning of skeletal muscle cells. It acts as a barrier, separating the genetic material in the nucleus from the cytoplasm, while also providing a scaffold for chromatin (Box 1) organization (Henderson et al., 2017). The nuclear lamina, situated just beneath the inner nuclear membrane, provides mechanical support to the nucleus by interacting with the inner nuclear membrane, chromatin and nuclear pore complexes (NPCs; Box 1). Lamins, such as lamin A/C (encoded by *LMNA*), are key proteins that connect the nuclear lamina to integral proteins within the inner nuclear membrane, such as emerin (encoded by *EMD*) and the trimeric SAD1/UNC84 (SUN) domain-containing proteins SUN1 and SUN2 (Heller et al., 2020; Henderson et al., 2017). These proteins span the inner nuclear membrane and bind to the tails of nesprins (encoded by SYNE genes) in the perinuclear space. Nesprins span the outer nuclear membrane to interact with actin, intermediate filaments or microtubules in the cytoplasm. The outer nuclear membrane is then continuous with the endoplasmic reticulum (ER), enabling it to play a role in the synthesis and transport of proteins and lipids. This structural connection spanning the nuclear envelope is referred to as the LINC complex (Heller et al., 2020). By connecting the nuclear interior with the cytoskeleton, the LINC complex facilitates the distribution of forces across the cell, which is particularly important in contractile muscle cells that endure mechanical stress. This complex also ensures the nucleus is correctly positioned within the cell and is involved in mechanotransduction. Mechanical forces induce changes in nuclear lamina polymerization, which impacts chromatin condensation, thereby altering transcriptional and translational efficiency, and allowing muscle to adapt to changes in physical activity (Jabre et al., 2021).

Mutations that impact the integrity of the nuclear envelope have been linked to several human diseases, including muscular dystrophies, premature aging syndromes, lipodystrophies, cardiomyopathies, cancer and a variety of neurological syndromes. EDMDs are commonly associated with abnormalities in the proteins that compose the nuclear envelope, a condition often referred to as ‘nuclear envelopathy’ (Heller et al., 2020). They are characterized by the clinical triad of joint contractures, progressive muscle weakness and atrophy, and cardiac involvement (Heller et al., 2020). In dominantly inherited forms of EDMD, joint contractures may appear after the onset of muscle weakness and tend to involve the elbows, ankles and posterior cervical muscles (Heller et al., 2020). Severe contractures can cause loss of ambulation by limitation of movement of the spine and lower limbs. Weakness and wasting typically start in a humero-peroneal distribution (Fig. 2D) and can later involve the scapular and pelvic girdle muscles. Cardiac involvement is usually apparent by the second to third decades of life and may include cardiomyopathies and cardiac conduction defects, putting patients at increased risk for cerebral emboli and sudden death (Heller et al., 2020). Muscle biopsies may show abnormal nuclear morphology as well as nonspecific myopathic and dystrophic changes (Heller et al., 2020).

Dominant mutations in *LMNA*, which encodes lamin A/C, can cause a wide variety of syndromes, including EDMD, dominantly inherited LGMD and dilated cardiomyopathy (Muchir et al., 2000). Demonstrating the importance of nuclear envelope function in extra-muscular tissues, dominant mutations can also cause disorders of peripheral nerve (type 2 Charcot-Marie-Tooth disease), adipose tissue (Dunnigan-type familial partial lipodystrophy) and a premature aging syndrome (Hutchinson-Gilford progeria syndrome) (Kang et al., 2018). *LMNA* mutations can display significant intra- and inter-family variability, with different members of the same family having an EDMD, LGMD or dilated cardiomyopathy phenotype. The molecular and biochemical pathogenesis by which mutations in *LMNA* lead to tissue-specific diseases remains unclear. It is interesting to note that recessive *LMNA* mutations can cause a severe EDMD, *Lmna* knockout in mice results in EDMD and dilated cardiomyopathy phenotypes, and haploinsufficient mice develop a later-onset cardiomyopathy (Chandar et al., 2010; Heller et al., 2020; Kang et al., 2018). These findings and other experimental data have led to the hypothesis that some *LMNA* point mutations cause disease via structural abnormalities of the nuclear membrane in a dominant-negative manner (Cattin et al., 2013; Kang et al., 2018; Perovanovic and Hoffman, 2018; Zwerger et al., 2013). There is also experimental support for disease mechanisms involving gain-of-function changes in heterochromatin formation at the nuclear membrane. This perturbs epigenomic imprinting during terminal differentiation of cells, leading to a mixture of gene silencing and activation at incorrect genomic loci (Perovanovic and Hoffman, 2018; Perovanovic et al., 2016; Solovei et al., 2013). Several preclinical therapeutic strategies have shown promise in laminopathies, including p38α mitogen-activated protein kinase (MAPK) inhibitors, mammalian target of rapamycin (mTOR) inhibitors and nicotinamide riboside, a precursor to nicotinamide adenine dinucleotide (NAD^+^) (Heller et al., 2020). Dominant mutations in the nuclear envelope proteins nesprin 1 and 2 (encoded by *SYNE1* and *SYNE2*, respectively), which traverse the outer nuclear membrane, can also cause EDMD phenotypes (Zhang et al., 2007). Nuclei from patient fibroblasts demonstrate structural abnormalities of nuclei, chromatin reorganization, and loss of nuclear envelope integrity with mislocalization of emerin and lamin A/C (Zhang et al., 2007). These changes were recreated following RNAi-mediated knockdown of *SYNE1* or *SYNE2* in normal fibroblasts, leading the authors to suspect that mutations have a dominant-negative effect on nuclear stability and uncoupling of the nucleoskeleton and cytoskeleton (Zhang et al., 2007).

*TMEM43* encodes the LUMA protein, an inner nuclear membrane protein that binds emerin, lamin A/C and SUN2, and plays a structural role in maintaining nuclear envelope shape. Mutations in *TMEM43* that cause an EDMD phenotype impact its oligomerization and are thought to have a dominant-negative impact on protein complex formation within the nuclear membrane (Liang et al., 2010, 2011).

## Nucleocytoplasmic shuttling

The nuclear envelope is also involved in a dynamic exchange between the nucleus and the cytoplasm known as nucleocytoplasmic shuttling (Box 1). Through NPCs, the nuclear membrane regulates the transport of molecules such as proteins and RNA in and out of the nucleus (Paci et al., 2021). This shuttling is essential for various cellular processes, including the response to growth signals and regulation of gene expression. Although small molecules (<40 kDa) may passively diffuse across NPCs, larger molecules require active shuttling that depends on nuclear transport receptors such as importins, which mediate entry into the nucleus, and exportins, which mediate exit. Importins and exportins bind their specific cargoes and facilitate their transit through NPCs, leveraging GTP hydrolysis inside the nucleus via the small RAS GTPase RAN (Box 1) (Paci et al., 2021). Abnormalities in nucleocytoplasmic transport have been implicated in neurodegenerative disorders, such as motor neuron disease, as well as muscular dystrophies.

Mutations in *TNPO3*, which encodes transportin-3, an importin critical for nucleocytoplasmic shuttling, were identified as the cause of LGMDD2 (Melià et al., 2013; Torella et al., 2013). Most of the identified *TNPO3* mutations cause a frameshift at the C-terminus, creating a 15- to 20-amino acid extension. Patients have a wide range of symptom onset, with a mean of 16 years. Individuals with earlier onset tend to have a more rapid course, progressing to being non-ambulatory by their second or third decade of life. Patients typically have symmetric proximal predominant weakness that spreads distally over time. Some have skeletal abnormalities such as long fingers, finger flexor contractures, pes cavus (Box 1) and scoliosis. About a third have dysphagia, while respiratory insufficiency and cardiac involvement are minimal. Myopathology shows typical chronic myopathic changes, rimmed vacuoles, aggregates, abnormalities in myofibrillar internal architecture, and inclusion bodies reactive for the autophagic adaptor protein P62 and the RNA-binding protein (RBP; Box 1) TDP43. Another distinct feature is abnormally large nuclei with central pallor. Transportin-3 belongs to the importin beta family and transports proteins into the nucleus, such as splicing factors. It is thought to function as a dimer and typically localizes to the nuclear envelope, stress granules (Box 1) and annulate lamellae, which are cytosolic NPCs (Costa et al., 2021, 2022; Melià et al., 2013). Disease-causing mutations appear to increase stability of the protein, but how they cause disease is not entirely clear (Costa et al., 2022). Interestingly, the replication cycle of human immunodeficiency virus (HIV) requires transportin-3 for the nuclear import of its integrase to facilitate integration of the HIV genome into the host cell. *TNPO3* mutations that cause LGMDD2 impair the ability of HIV to replicate, resulting in patients' lymphocytes being resistant to HIV infection (Fricke et al., 2013; Rodríguez-Mora et al., 2019).

## Epigenetic misregulation of transcriptional activity

FSHD is a complex genetic muscle disorder characterized by progressive, often asymmetric muscle weakness and atrophy starting in the face, shoulder girdle and upper arm muscles, followed by involvement of the trunk, pelvic girdle and distal anterior compartment leg (Fig. 2C). Onset is commonly between 15 and 30 years of age, but ranges from infancy to late adulthood (Lemmers et al., 2012; Tawil et al., 1998; van den Boogaard et al., 2016). Approximately 20% of patients eventually become wheelchair dependent. Two genetic forms of FSHD exist: FSHD type 1 (FSHD1) and FSHD type 2 (FSHD2). They share similar clinical presentations and a common downstream mechanism that causes muscle cell death, namely, the misexpression of the double homeobox 4 (*DUX4*) gene, which is normally epigenetically repressed in most somatic tissues (Lemmers et al., 2012; Tawil et al., 1998; van den Boogaard et al., 2016).

FSHD1 is the most common form of FSHD and accounts for ∼95% of cases. FSHD1 is linked to a genetic deletion on chromosome 4q35. This deletion reduces the number of D4Z4 macrosatellite repeats to 1-10 units from the typical 8-100 found in unaffected individuals (Tawil et al., 1998). Each D4Z4 unit of the repeat array contains a copy of the *DUX4* gene, which is normally expressed in the testis and silenced in somatic tissue. Contraction of the D4Z4 repeats leads to a relaxed chromatin structure and incomplete epigenetic repression (Box 1) of DUX4 expression in myonuclei, which is toxic to muscles. Highly homologous D4Z4 repeat arrays exist at multiple chromosomal locations (4qA, 4qB, 4q10). However, stable *DUX4* transcripts can only be produced from the 4qA chromosomal region as the last repeat unit is followed by a third exon containing a polyadenylation signal (PAS; Box 1) that stabilizes the *DUX4* transcript from the last repeat unit (Tawil et al., 1998).

FSHD2 is caused by mutations in chromatin modifier genes (Box 1), most often *SMCHD1* and sometimes DNA methyltransferase 3 beta (*DNMT3B*) or ligand-dependent nuclear receptor-interacting factor 1 (*LRIF1*) (Lemmers et al., 2012; van den Boogaard et al., 2016). Unlike FSHD1, somatic de-repression of *DUX4* expression in FSHD2 patients is due to D4Z4 chromatin relaxation without a D4Z4 repeat contraction below 10. However, FSHD2 patients do tend to have shorter D4Z4 repeat arrays (0-11 repeats) than average. Over 85% of FSHD2 patients have heterozygous, loss-of-function mutations in SMCHD1 (structural maintenance of chromosomes flexible hinge domain containing 1), a chromatin repressor involved in the establishment and maintenance of CpG methylation at specific loci, including at D4Z4 repeats. SMCHD1 haploinsufficiency leads to hypomethylation of the D4Z4 repeats, relaxing the chromatin structure at this region. Other causes for FSHD2 include heterozygous mutations in *DNMT3B* and recessive mutations in *LRIF1* gene. *DNMT3B* is a known D4Z4 chromatin remodeler, and dominant point mutations were associated with hypomethylation of this region. Interestingly, FSHD1 disease severity can be impacted by mutations in these chromatin modifier genes.

A variety of therapeutic strategies for FSHD are being investigated. Losmapimod, a small-molecule inhibitor of p38α/β MAPK was identified in a drug screen as an inhibitor of DUX4 expression (Campbell et al., 2017). A phase 2 clinical trial showed reduced fat infiltration of muscle on magnetic resonance imaging, and improved multiple clinical outcome measures, without modifying DUX4-driven gene expression (Jagannathan et al., 2022). A multicenter phase 3 trial was recently completed and failed to meet its primary endpoint. A key difference from the phase 2 study was that patients receiving placebo did not show a decline in functional status. Other approaches involve targeting *DUX4* mRNA for degradation using RNAi (Table 2) (Chen et al., 2016a; Wallace et al., 2011, 2018). A variety of strategies using CRISPR/Cas9 (Box 1) have also been tested, including CRISPR interference (CRISPRi) targeting the *DUX4* promoter or exon 1 to silence *DUX4* expression, or CRISPR-Cas9 base editing for mutagenesis of the 4qA PAS, preventing stabilization of *DUX4* transcripts (Table 2) (Himeda et al., 2021; Šikrová et al., 2021).

## RNA related

### Repeat expansion disorders

Skeletal muscle disorders caused by repeat expansion mutations represent a unique group of genetic conditions. Disorders in which the expansion of specific nucleotide repeats is within non-coding regions of genes may cause disease via toxicity directly from RNA foci (Box 1). These aggregates of mutant RNA accumulate in nuclei and sequester various RBPs, disrupting the processing of other RNAs, including those involved in muscle function and maintenance. The specific genes involved, the location and type of the repeats, and the proteins affected by the RNA aggregates largely determine the distinct phenotypes of each condition. Disease may also be related to repeat-associated non-ATG translation (Box 1), which produces toxic peptides from the repeat expansion. When the repeat expansion is within coding regions of genes, disease may also be caused via translation of proteins with long runs of the same amino acid, leading to its aggregation and potential cellular toxicity.

Myotonic dystrophy type 1 (DM1) is caused by an expanded CTG repeat in the 3’ untranslated region (UTR) of the dystrophia myotonica-protein kinase (*DMPK*) gene (Brook et al., 1992). DM1 can manifest anytime from infancy to adulthood. The hallmark feature is myotonia – difficulty relaxing muscles after contraction. Muscle weakness typically begins in the face, neck and distal limb muscles, and slowly progresses proximally. Individuals have a characteristic appearance with frontal balding, ptosis (droopy eyelids), and atrophy of the temporalis, masseter and other facial muscles (Johnson, 2019; Soltanzadeh, 2022). Other symptoms include cataracts, cardiac conduction defects, endocrine disorders, and central nervous system involvement such as cognitive and behavioral impairment and central sleep disorders. Life expectancy can be reduced, especially with cardiac and respiratory complications. Histopathologically, there is evidence of myopathic changes and, occasionally, type 1 fiber smallness and predominance, with ring fibers and increased internal nuclei, often with several per muscle fiber, occurring in clumps or longitudinal chains. In unaffected individuals, the 3′ UTR of *DMPK* typically contains 5-35 repeats. In DM1 patients, it expands to hundreds or thousands of repeats, with longer repeats correlating with earlier onset and increased disease severity. The phenomenon of genetic anticipation is seen in this disease, where the CTG repeat expansion is unstable, leading to its expansion in successive generations, causing an earlier onset of disease and a more severe phenotype (Soltanzadeh, 2022). This expanded repeat in the RNA forms hairpin structures that accumulate in the nucleus, leading to a toxic effect. These RNA aggregates sequester specific RBPs, such as muscleblind-like (MBNL) proteins, disrupting their normal functions in RNA splicing (Box 1), localization and stability. The resultant splicing abnormalities affect various pre-mRNAs, leading to the diverse symptoms of DM1 (Johnson, 2019). A number of therapeutic approaches are currently under investigation and are aimed at selectively knocking down the expanded RNA, modulating the RBPs (MBLN1) that are sequestered in RNA foci, and pharmacologically targeting downstream maladaptive signaling pathways (GSK3B, PKC, AMPK) (Table 2) (Soltanzadeh, 2022).

Myotonic dystrophy type 2 (DM2) is caused by a CCTG repeat expansion in the first intron of *CNBP* [encoding zinc finger (ZFN)9] (Day et al., 2003). Unlike DM1, the repeat in DM2 is located in an intronic region and typically ranges from 75 to more than 11,000 repeats. Similar to DM1, the expanded repeat RNA forms foci that sequester RBPs such as MBNL, leading to splicing abnormalities. However, the pattern of mis-spliced genes differs slightly between DM1 and DM2, potentially contributing to some differences in clinical presentation (Johnson, 2019; Soltanzadeh, 2022). Also, although loss of the *DMPK* gene in DM1 does not appear to cause any untoward effects, early evidence indicates that loss of the *CNBP* gene may cause muscle toxicity as well; therefore, loss of the encoded ZFN9 protein function may contribute to the pathogenesis. DM2 generally presents later than DM1, often in the third or fourth decade. Clinical features include myotonia, muscle pain and weakness primarily in proximal muscles, contrasting with the distal onset in DM1. DM2 patients similarly experience cataracts, cardiac abnormalities and diabetes, but typically with a milder course than that of DM1 patients. DM2 muscle histopathology is typically less severe than in DM1, and the increase in internal nuclei tends to occur in type 2 muscle fibers. The progression of DM2 is generally slower than that of DM1, and the life expectancy is less affected. Additionally, repeat length does not correlate with disease severity, and genetic anticipation does not appear to occur.

Oculopharyngeal muscular dystrophy (OPMD) results from a trinucleotide repeat expansion in the *PABPN1* gene (Banerjee et al., 2013; Brais et al., 1998). The disease usually presents in the fifth to sixth decade of life. The primary symptoms are ptosis and dysphagia, with later involvement of limb muscles (Fig. 2E). Unlike DM1 and DM2, OPMD does not typically cause myotonia or systemic features. Myopathology shows intranuclear inclusions and rimmed vacuoles. Disease progression is slow, and life expectancy is often normal, but the quality of life is significantly affected by swallowing difficulties and vision impairment. The *PABPN1* gene encodes poly(A) binding protein nuclear 1, which controls the poly(A) tail length on RNA transcripts and alternative polyadenylation site usage, thus affecting mRNA levels and stability. The normal allele contains 10 alanine-encoding GC(N) repeats [(GCG)6(GCA)3(GCG)1], whereas the mutant allele contains an expansion of 11-18 GC(N) repeats (typically GCG) (Banerjee et al., 2013). The repeat expansion is within a coding region, resulting in PABPN1 protein with an expanded polyalanine tract, leading to the formation of insoluble aggregates in the nucleus. These inclusions sequester other important nuclear proteins, possibly disrupting mRNA processing, nuclear export and protein quality control systems (Yamashita, 2021). Thus, the mechanism is believed to be related to toxicity from the mutant protein rather than RNA toxicity. Therapies that reduce protein aggregation have shown promise in several preclinical studies in cell, fly and mouse OPMD models. The disaccharide trehalose, which reduces aggregation of mutant polyQ/A proteins, advanced to clinical trials for OPMD and showed some improvement in dysphagia (Argov et al., 2016). Interestingly, other potential therapies, such as lithium chloride, valproic acid and sirtinol, reduced the cell death without reducing protein aggregation (Yamashita, 2021). Knockdown-and-replace gene therapy approaches (Fig. 4D and Box 2) showed promise in preclinical studies and are now moving into a phase 1b/2a clinical trial (Table 2) (Abu-Baker et al., 2019; Malerba et al., 2017). The knockdown-and-replace strategy is necessary as absence of *Pabpn1* in mice is embryonic lethal, and knockdown of *Pabpn1* in adult mice induces myodegeneration (Malerba et al., 2017).

Oculopharyngodistal myopathy (OPDM) is a group of disorders caused by mutations in several genes such as *LRP12*, *GIPC1*, *NOTCH2NLC* and *RILPL1* (Deng et al., 2020; Ishiura et al., 2019; Yu et al., 2022). All four OPDM disease-causing genes share the same repeat motif (CGG) and the same mutation location (5′ UTR), suggesting that these different genes share a common pathomechanism. How a presumably untranslated CGG repeat leads to myodegeneration is unclear. One possible pathomechanism is similar to that seen in DM1 and DM2, whereby toxic RNA foci form from the repeat expansion and sequester RBPs (Kumutpongpanich et al., 2021). Another is an RNA-dependent gain-of-function mechanism similar to that described in fragile X-associated tremor/ataxia syndrome (FXTAS; Box 1), by which repeat associated non-ATG (RAN) translation from the CGG repeat produces a toxic poly-glycine protein (Cleary et al., 2018; Li et al., 2023; Yu et al., 2022). Individuals with OPDM present with progressive ptosis, ophthalmoparesis, facial and masseter weakness, dysphagia, and muscle weakness of distal limbs (Fig. 2F). Myopathology demonstrates chronic myopathic changes with rimmed vacuoles and filamentous intranuclear inclusions. Pure OPDM phenotypes have been associated with CGG repeat expansions in *RP12*, *GIPC1* and *RILPL1*. CGG repeat expansions in *NOTCH2NLC* cause OPDM with additional central and peripheral nervous system features, such as retinal degeneration, ataxia, tremor, deafness, peripheral neuropathy and leukoencephalopathy on brain imaging (Ogasawara et al., 2020).

### RBPs

Several dominantly inherited disorders with skeletal muscle involvement are associated with mutations in RBPs. These proteins have RNA recognition domains (RRMs) to facilitate RNA binding. They participate in all steps of the mRNA cycle, including transcription, maturation, nucleocytoplasmic transport, translation, stability, degradation and sequestration (Picchiarelli and Dupuis, 2020). Many RBPs associated with neuromuscular diseases are part of a large group of proteins collectively termed heterogeneous nuclear ribonucleoproteins (HNRNPs), which contain low complexity domains that resemble yeast prions and are called ‘prion-like domains’ (PrLDs; Box 1) (Picchiarelli and Dupuis, 2020). PrLDs have prion-like properties, meaning that they can undergo conformational changes and form reversible aggregates. They are rich in uncharged polar amino acids (asparagine, glutamine and tyrosine) and glycine, enabling them to undergo liquid-liquid phase separation (LLPS; Box 1), a process that allows the formation and maintenance of membrane-less organelles like cytoplasmic stress granules, or key nuclear subdomains such as nucleoli, paraspeckles and P-bodies (Lee et al., 2022). These dynamic, reversible and stress-responsive compartments allow specific biochemical processes, particularly those involving RNA (like splicing or transport), to occur efficiently. RBPs also shuttle between the nucleus and cytoplasm, facilitating the proper distribution and function of RNAs within the cell.

Muscle disorders resulting from abnormalities in RBPs are increasingly understood in the context of their PrLDs and the phenomenon of LLPS, as well as abnormalities in shuttling and their mislocalization from the nucleus to the cytoplasm. RBPs, such as HNRNPDL, HNRNPA2B1, HNRNPA1, TDP43 and TIA1, have PrLDs that contribute to pathogenic mechanisms when mutated. Under stress, RBPs typically undergo LLPS to form reversible, dynamic aggregates such as stress granules, which are considered to be protective. However, disease-causing mutations are thought to render this process pathological, leading to impaired LLPS, and persistence of stress granules or abnormal, irreversible, toxic aggregates (Nussbacher et al., 2019). This toxicity may be related to aggregates sequestering RNA and other RBPs, leading to a loss of function in critical RNA-processing pathways. This impairment can have widespread effects, particularly in muscle and nerve cells that are heavily dependent on precise RNA regulation. Mutations in RBPs can also impair their nucleocytoplasmic shuttling, leading to their accumulation in the wrong cellular compartment (cytoplasm), and disrupting the delicate balance of RNA processing and localization (Nussbacher et al., 2019). Two common hypotheses for disease mechanisms involve a gain of toxicity from RBPs mislocalizing to the cytoplasm, leading to abnormal binding and processing of cytosolic RNA targets, and/or nuclear loss of function, leading to lack of proper RNA processing and splicing.

Dominant mutations in the RBP genes *HNRNPA1* and *HNRNPA2B1* are linked to multisystem proteinopathies (MSPs). This heterogeneous group of disorders is characterized by prominent proteinaceous pathology involving an interconnected axis of tissues: bone, muscle, motor neuron and brain (Kim et al., 2013). MSP is most commonly associated with inclusion body myopathy (IBM), Paget disease of bone (PDB), frontotemporal dementia (FTD), amyotrophic lateral sclerosis (ALS) or various combinations of these disorders. There is also significant intrafamilial variability in the tissues affected (Kim et al., 2013). The previously used term, inclusion body myopathy associated with PDB and frontotemporal dementia (IBMPFD), is no longer used as several other phenotypes can be seen, including motor neuron disease, Parkinson's disease, hereditary spastic paraplegia and neuropathy. The myopathy in MSP is typically characterized by slowly progressive weakness and atrophy of proximal and distal muscles of the upper and lower extremities, foot drop and scapular winging. Muscle pathology is notable for cytoplasmic aggregates, inclusions and rimmed vacuoles containing proteins such as TDP43 and P62. Muscle biopsy will frequently show mixed myopathic and neurogenic findings, suggesting a co-existent motor axonopathy. HNRNPA1 and HNRNPA2, the shorter and more prevalent of the two spliced isoforms of HNRNPA2B1 (HNRNPA2, HNRNPB1), have similar domain architecture: two N-terminal RRMs and a C-terminal PrLD containing a PY-nuclear localization sequence (NLS; Box 1) that mediates nuclear import (Kim et al., 2013). MSP-linked mutations involve a valine substitution at a conserved aspartate residue in the PrLD. Both HNRNPA1 and HNRNPA2 can form amyloid fibrils *in vitro* that are self-seeding, meaning that they can nucleate the aggregation of soluble protein. Disease-associated mutations greatly accelerate fibrillization. *In vitro*, the mutant proteins can seed their own amyloid fibril assembly and the assembly of the corresponding WT protein, providing a possible explanation for dominant inheritance. Muscle histopathology demonstrates mislocalization of mutant HNRNPA1 and HNRNPA2 from the nucleus to cytoplasmic stress granules and aggregates (Kim et al., 2013). Disease mutations are thought to cause a toxic or gain of function, as knockdown of *HNRNPA2B1* and the disease-causing mutation, p.D290V, cause entirely different downstream alternative splicing events (Hutten and Dormann, 2016; Martinez et al., 2016). Given the interconnected axis of tissues involved in MSP, it is enticing to envision a disease mechanism involving prion-like proteopathic spread of toxic aggregates from one tissue to another. This has yet to be demonstrated. Recently, dominant frameshift mutations in *HNRNPA2B1* were found to be associated with an early-onset OPMD phenotype. All disease-causing frameshift mutations abolished the stop codon, creating HNRNPA2B1 proteins with an extension of their C-terminal sequence. This reduces its affinity for the nuclear import receptor karyopherin β2, altering its nucleocytoplasmic transport dynamics, leading to cytoplasmic accumulation of HNRNPA2 (Kim et al., 2022).

Dominant mutations in the PrLD of the RBP T-cell-restricted intracellular antigen-1 (TIA1) cause several phenotypes including ALS, FTD and a rimmed vacuolar myopathy, called Welander distal myopathy (WDM). The myopathy is characterized initially by finger and wrist extensor weakness around age 50, and later involves finger flexors and toe and ankle extensors (Hackman et al., 2013). Disease progression is slow, and patients typically remain ambulatory. In the nucleus, TIA1 plays a significant role in alternative splicing of certain pre-mRNAs. In the cytoplasm, TIA1 is a key component of stress granules, which are involved in the regulation of RNA-translation metabolism. Stress granules prevent the translation of some mRNAs and favor the translation of those that give rise to proteins that help to overcome stress and recover. Similar to mutations in other RBPs, such as HNRNPA1, HNRNPA2B1 and TDP43, mutations affecting the PrLD of TIA1 alter its LLPS properties, enhance its fibrillization and impair stress granule clearance (Hackman et al., 2013; Lee et al., 2018; Mackenzie et al., 2017). TIA1 mutations may cause disease via a toxic gain-of-function mechanism, as the mutated TIA1 protein abnormally interacts with RNA and other proteins and does not inhibit normal TIA1 protein function (Fernández-Gómez et al., 2022). Rare patients with homozygous *TIA1* mutations have a more severe disease with earlier onset, distal and proximal muscle involvement, and faster progression, eventually leading to loss of ambulation (Hackman et al., 2013).

HNRNPDL is an RBP with two PrLDs. Its three isoforms are generated via alternative splicing, and they lack none, one or both PrLDs. Multivalent interacting residues (Arg and Tyr) are spatially separated in the two PrLDs, and their presence or absence dictates the HNRNPDL LLPS and shuttling properties (Batlle et al., 2020). Currently, two-point mutations, affecting the same aspartate (p.D378N or H) in the C-terminal PrLD of HNRNPDL, are known to definitively cause LGMDD3 (Vieira et al., 2014). This mutation impacts the ability of hnRNPDL to undergo LLPS, accelerating its aggregation and reducing its solubility in the muscle of *Drosophila*. A genetic loss-of-function mechanism was hypothesized based on mutant hnRNPDL precipitation into insoluble aggregates in a *Drosophila* model, absence of cytoplasmic inclusions in patient muscle biopsies and a *Drosophila* model, and Hnrnpdl knockdown in zebrafish embryos that results in uncoordinated movements and disorganized myofibers 4 days post-fertilization (Batlle et al., 2020; Vieira et al., 2014). However, muscle histopathology in LGMDD3 patients does show cytoplasmic bodies, P62 accumulations, rimmed vacuoles, autophagic vacuoles with sarcolemmal features (AVSF; Box 1) and cytoplasmic areas with tubulofilamentous inclusions on electron microscopy (Berardo et al., 2019). Additionally, mutations involving the equivalent aspartate residue within the PrLD of HNRNPA1 and HNRNPA2B1 cause MSP via a suspected toxic gain of function (Harrison and Shorter, 2017). Overall, it is not yet clear whether these mutations cause disease via a toxic gain of function potentially from misfolded RBPs, a loss of function or a combination of these non-mutually exclusive possibilities. Clinically, LGMDD3 is characterized by a later onset and a wide spectrum of phenotypes, including a limb-girdle phenotype and distal limb weakness affecting the flexor muscles of fingers and toes, with some individuals having a scapuloperoneal pattern of weakness (Berardo et al., 2019). There are several potentially distinguishing features including cataracts, finger contractures and diabetes. One family has been reported to have cognitive impairment, which is notable as mutations in other HNRNPs cause MSPs associated with FTD. Recently, two nearby mutations in the PrLD of HNRNPDL (p.Y377C, p.G373R) were identified in singleton patients with rimmed vacuolar myopathies (Cotta et al., 2023; Ibaceta et al., 2023). Interestingly, the p.Y337C mutation only caused disease in a homozygous state, as the parents and a sibling who were heterozygous were unaffected. This patient had an early onset of disease, reminiscent of the more severe phenotype associated with homozygous mutations in the RBP TIA1 (Hackman et al., 2013). Similar to the p.D378 mutations in HNRNPDL, p.Y377C had an increased propensity to form stress granules compared to WT at baseline and following stress when overexpressed in cells (Ibaceta et al., 2023).

*TDP43* encodes transactive response DNA-binding protein 43, which binds single-stranded DNA and RNA. It primarily localizes to the nucleus but also shuttles to the cytoplasm and may regulate various steps of RNA biogenesis (Tziortzouda et al., 2021). TDP43-positive inclusions are a key neuropathological finding in FTD and ALS (Arai et al., 2006; Neumann et al., 2006). Pathogenic missense variants in its C-terminal PrLD cause ALS and modulate its LLPS and aggregation properties (Kim et al., 2020). TDP43-positive inclusions are a common feature of rimmed vacuolar myopathies (Küsters et al., 2009). TDP43 can also form myo-granules that contain mRNAs encoding sarcomeric proteins and localize to sites of newly forming sarcomeres (Vogler et al., 2018). Myo-granules are normally cleared in mature myofibers but become abnormally increased in pathological muscle tissues undergoing repeated rounds of degeneration and regeneration. This may enhance formation of pathological TDP43 aggregates and amyloid fibrils, disrupting muscle structure and function. Recently, a five-generation family with a dominantly inherited rimmed vacuolar myopathy was identified to have a frameshift mutation in TDP43, producing a C-terminally altered PrLD (p.Trp385IlefsTer10) (Pereira et al., 2023). Muscle histopathology demonstrated TDP43-positive sarcoplasmic inclusions and accumulations of autophagosomes. This mutant TDP43 does not form liquid-like condensates but does have increased aggregation propensity. In *Drosophila*, the mutant TDP43 was able to rescue a neurodevelopmental lethal null phenotype. It also reduced toxic gain-of-function properties upon overexpression, suggesting a disease mechanism involving partial loss of function (Pereira et al., 2023).

*ANXA11* encodes a calcium-dependent phospholipid-binding protein that acts as a tether between RNA granules and lysosomes. It possesses an N-terminal low-complexity domain, facilitating its phase separation into RNA granules, and a C-terminal membrane-binding domain, enabling interactions with lysosomes (Johari et al., 2022; Leoni et al., 2021). A mutation in its N-terminal low-complexity domain (p.D40Y) was recently found to cause a dominantly inherited late-onset myopathy with rimmed vacuoles, annexin A11 accumulations, myofibrillar abnormalities and subsarcolemmal autophagic material. A mutation at this same residue was previously reported in ALS patients (p.D40G) (Johari et al., 2022; Leoni et al., 2021). ANXA11 mutations associated with ALS alter its LLPS properties and lysosomal interactions (Liao et al., 2019). They are thought to contribute to disease via loss of function, as ANXA11 knockdown similarly impaired mRNA transport to distal portions of neurons, and also potentially via a toxic gain of function owing to protein aggregates sequestering additional RNA granule proteins and impacting RNA metabolism (Liao et al., 2019).

Matrin 3 is a major component of the nuclear matrix and functions as an RBP with one zinc finger domain, two RRMs and two large PrLDs (Malik and Barmada, 2021). Matrin 3 is similar to other RBPs with PrLDs for which mutations are linked not only to ALS and FTD but also to skeletal muscle involvement (Chompoopong et al., 2023; Malik and Barmada, 2021; Müller et al., 2014). *MATR3* mutations were first associated with an autosomal-dominant distal myopathy with vocal cord paralysis and pharyngeal weakness [vocal cord pharyngeal distal myopathy (VCPDM)] (Feit et al., 1998; Müller et al., 2014). The causative mutation, p.S85C, affects a highly conserved residue within the N-terminal PrLD. Muscle histopathology is notable for rimmed vacuoles, and immunostaining reveals mislocalized cytoplasmic matrin 3 and myofiber inclusions reactive for TDP43, P62/SQSTM1 and ubiquitin. Other cohorts affected by the same mutation had a slightly different phenotype, with distal weakness without pharyngeal weakness or vocal cord paralysis, and later developed weakness of axial, respiratory and proximal muscles (Müller et al., 2014). Mutant matrin 3 accumulates in detergent-insoluble aggregates and may interrupt physiological LLPS, eventually leading to irreversible toxic RBP aggregates characteristic of other myo- and neuro-degenerative disorders associated with RBP mutations (Malik and Barmada, 2021; Sprunger et al., 2022).

## Protein homeostasis

The importance of protein homeostasis in skeletal muscle is clearly illustrated by the several myopathies caused by mutations in protein chaperones, termed chaperonopathies (Box 1), as well as proteins involved in turnover pathways such as the ubiquitin-proteasome system (UPS), autophagolysosomal (APL) system (Box 1) and proteases (Carlisle et al., 2017; Dowling et al., 2021; Kitajima et al., 2020; Sarparanta et al., 2020). Protein quality control is thought to be particularly crucial for skeletal muscle owing to its large proteostatic burden from constant mechanical stress and the associated misfolding, damage and re-synthesis of abundant amounts of structural myofibrillar proteins. Many of these diseases are characterized histopathologically by vacuoles, aggregates and myofibrillar abnormalities.

### Chaperonopathies

Chaperones (Box 1) are central to protein quality control. They facilitate the folding of their client proteins into their native conformation, prevent client protein misfolding and aggregation, and direct damaged, misfolded or aggregated proteins to turnover pathways (Carlisle et al., 2017; Sarparanta et al., 2020). Many chaperones are called heat shock proteins (HSPs; Box 1) as expression of this group of proteins was initially found to be inducible following heat stress (Kampinga et al., 2009).

*CRYAB* encodes αB-crystallin, a small heat shock protein also known as HSPB5. A key role of small heat shock proteins (HSPBs) is to function as ‘holdases’, preventing partly folded proteins from aggregating (Carra et al., 2019; Sarparanta et al., 2012). αB-crystallin is a structural protein in the lens of the eye and is expressed following stress at particularly high levels in skeletal and cardiac muscle (Inaguma et al., 1995; Larkins et al., 2012; Sarparanta et al., 2020). It has a sarcoplasmic localization at baseline and, following stress (stretch, eccentric contraction), it translocates to bind, stabilize and prevent aggregation of its client proteins, desmin at the Z-disc and titin at the I-band (Golenhofen et al., 1998, 2002). Dominant missense and truncating variants in *CRYAB* cause a myofibrillar myopathy with clinical and histopathological similarities to patients with dominant mutations in its client protein, desmin (Selcen and Engel, 2003; Vicart et al., 1998). Patients have progressive distal predominant weakness, sometimes with proximal involvement, respiratory insufficiency and cardiomyopathy (Selcen and Engel, 2003; Vicart et al., 1998). Muscle biopsies demonstrate features typical of myofibrillar myopathies, with vacuoles and subsarcolemmal aggregates reactive for desmin and αB-crystallin (Schröder and Schoser, 2009). A key difference from desminopathies is the presence of cataracts, which is attributable to αB-crystallin's expression in the lens. Recessively inherited truncating frameshift mutations cause a fatal infantile-onset myofibrillar myopathy (Bigio et al., 2011). Pathomechanistically, mutations typically impact its dimer interface or client-binding site and may have a dominant-negative effect on oligomeric αB-crystallin complexes, limiting their ability to prevent client protein aggregation (Dimauro et al., 2018; Sarparanta et al., 2012). Specifically, dominant missense mutations cause increased αB-crystallin binding to desmin and other intermediate filaments, promoting their interaction and leading to their aggregation (Perng et al., 1999, 2004). Additionally, mutations impair αB-crystallin binding to titin's spring elements, disrupting their extensibility (Zhu et al., 2009). Mutant αB-crystallin is also unstable and prone to aggregating, potentially contributing to disease via a loss of function (Dimauro et al., 2018; Vicart et al., 1998; Zobel et al., 2003).

Dominant mutations in BCL2-associated athanogene 3 (*BAG3*) cause a rapidly progressive childhood- or adolescence-onset myofibrillar myopathy with limb and axial weakness, peripheral neuropathy, respiratory failure and a severe dilated cardiomyopathy (Selcen et al., 2009). Muscle biopsies demonstrate myofibrillar disarray, rimmed vacuoles, and aggregates reactive for BAG3, αB-crystallin, HSPB8, desmin, filamin C and others (Selcen et al., 2009). BAG3 is a stress-inducible co-chaperone with high expression levels at the Z-disc in skeletal muscle. It interacts with small HSPs and HSP70s to stimulate macroautophagy, leading to lysosomal degradation of misfolded proteins. This process relies on DNAJ and HSPB proteins to deliver misfolded clients to HSP70s, and nucleotide exchange factors (NEFs) like BAG3 facilitate client protein release (Meister-Broekema et al., 2018; Sarparanta et al., 2020). In muscle, these interactions facilitate the targeting of misfolded filamin-C for degradation via the chaperone-assisted selective autophagy (CASA) pathway (Box 1) (Ulbricht et al., 2013a,b). Mutant BAG3 still binds HSP70, but is thought to impair client protein processing, causing a toxic gain of function via sequestration of itself, HSP70, client proteins and other associated HSPs into insoluble aggregates, leading to a collapse of protein quality control (Meister-Broekema et al., 2018). In fact, genetic or pharmacological inhibition of the interaction between mutant BAG3 and HSP70 corrects *in vitro* protein aggregation phenotypes (Meister-Broekema et al., 2018). Similar findings have been described in zebrafish in which knockdown of Bag3 leads to myofibrillar disintegration from impaired macroautophagic activity, whereas dominant Bag3 mutations lead to protein aggregates that sequester both WT and mutant Bag3, leading to myofibrillar abnormalities (Ruparelia et al., 2014). Interestingly, metformin, a drug used for treating diabetes, was identified via a screen as an autophagy-promoting compound capable of reducing Bag3 protein aggregates, and rescuing myofibrillar disintegration and swimming deficits in zebrafish (Ruparelia et al., 2021).

Mutations in *HSPB8* (also called *HSP22*) cause a variety of hereditary neuropathies (Irobi et al., 2004; Tang et al., 2005). More recently, dominant mutations in *HSPB8* were found to cause a distal predominant myopathy, sometimes with involvement of axial muscles, and often with a distal neurogenic component (Al-Tahan et al., 2019; Echaniz-Laguna et al., 2017; Ghaoui et al., 2016). On muscle biopsy, typical myofibrillar myopathy changes are seen, including rimmed vacuoles and aggregates reactive for myotilin, αB-crystallin, dystrophin, HSPB8, DNAJB6, myotilin, BAG3, TDP43 and ubiquitin. HSPB8 is involved in CASA, which in muscle is thought to maintain the cytoskeleton by degrading damaged components (Arndt et al., 2010; Ulbricht et al., 2013b). A homozygous knock-in mouse model with the neuropathy-associated p.K141N mutation develops a motor neuropathy and myopathy phenotype with myofibrillar myopathy features, including rimmed vacuoles and aggregates. Heterozygous mice have no functional deficits, but have similar pathological changes (Bouhy et al., 2018). *Hspb8* knockout mice do not develop a neuropathy, myopathy or myofibrillar pathology, suggesting that the p.K141N mutation exerts a dominant toxic effect (Bouhy et al., 2018). Two HSPB8 mutations – p.Q170Gfs*45 and p.P173Sfs*43 – have been reported in neuromyopathy patients and appear to preferentially affect muscle. They cause a 40-60% decrease in HSPB8 protein in patient muscles, with no sign of expression of the extended mutant protein (Al-Tahan et al., 2019; Echaniz-Laguna et al., 2017). Some have therefore suggested a haploinsufficiency mechanism, and a toxic or gain-of-function mechanism remains a possibility based on the *Hspb8* knockout mouse data (Al-Tahan et al., 2019; Bouhy et al., 2018; Echaniz-Laguna et al., 2017; Sarparanta et al., 2020). Additionally, dominant mutations in the small heat shock proteins HSPB1 and HSPB3, which typically cause distal hereditary motor neuropathies and other multisystem disorders, have in some cases been associated with myopathic features (Tedesco et al., 2022).

Dominant missense mutations in the HSP70 co-chaperone, DNAJB6, cause LGMDD1 (Box 3) (Harms et al., 2012; Sarparanta et al., 2012). Disease onset ranges from early childhood to late adulthood (Findlay et al., 2022). Most mutations cause proximal predominant weakness, while others are more commonly associated with distal weakness (Findlay et al., 2022). Muscle histopathology demonstrates aggregates, vacuoles and myofibrillar abnormalities (Sandell et al., 2016). HSP70 chaperones are a central hub of the protein quality control network, participating in many ATP-dependent protein-folding processes, whereas J-domain co-chaperones such as DNAJB6 target specific client proteins to HSP70s (Abayev-Avraham et al., 2023; Fan et al., 2003; Karamanos et al., 2019). Similar to other J-domain proteins, the alpha-helical N-terminal J-domain of DNAJB6 directly interacts with HSP70 and stimulates its ATPase activity, leading HSP70 to bind the recruited client with high affinity (Abayev-Avraham et al., 2023; Kityk et al., 2018). Adjacent to the J-domain is the glycine/phenylalanine (G/F) domain, which is thought to participate in auto-inhibition of the J-domain's ability to bind and activate HSP70 ATPase activity (Abayev-Avraham et al., 2023; Fan et al., 2003; Karamanos et al., 2019). Next is the serine/threonine (ST) low-complexity region, which is essential for DNAJB6 oligomerization and its ability to prevent protein aggregation (Abayev-Avraham et al., 2023; Månsson et al., 2014; Söderberg et al., 2018). Lastly, the C-terminal domain is critical for substrate binding (Abayev-Avraham et al., 2023; Karamanos et al., 2019). DNAJB6 has many different functions, including prevention of aggregation of polyglutamine-containing proteins, assisting CASA, suppression of malignant transformation of cancer, and facilitation of several viral replication cycles, such as HIV (Meng et al., 2016; Sarparanta et al., 2020). DNAJB6 is ubiquitously expressed in all tissues, yet dominant mutations selectively cause a myodegenerative disorder (Hageman et al., 2010; Seki et al., 1999). Absence of *Dnajb6* is embryonic lethal in mice through aggregation of a couple of DNAJB6 client proteins, keratin 8 and 18, causing failure of chorioallantoic fusion (Box 1) during placental development (Watson et al., 2007). DNAJB6 haploinsufficiency may also be deleterious as mice develop cardiac conduction abnormalities (Ding et al., 2022). Similar to DNAJB6 knockout, haploinsufficiency causes a phenotype different from the LGMD associated with dominant disease-causing mutations. DNAJB6 has two key isoforms: DNAJB6a is a larger nuclear predominant isoform and DNAJB6b is a shorter isoform that localizes to sarcomeric structures, including the Z-disc, which is a key site of pathology seen in muscle biopsies from patients (Bengoechea et al., 2015). All known mutations that cause LGMDD1 impact both isoforms and are localized to DNAJB6 J- or G/F domains (Findlay et al., 2022). Overexpression of mutant DNAJB6b, but not DNAJB6a, in mouse and zebrafish skeletal muscle causes myopathic changes, suggesting an isoform-specific component to disease (Bengoechea et al., 2015; Sarparanta et al., 2012). However, DNAJB6a is likely not completely dispensable in muscle, as a patient with a myofibrillar myopathy was found to have recessively inherited frameshift mutations selectively affecting DNAJB6a expression, and a knock-in mouse model recapitulated some of their phenotypic abnormalities (Qian et al., 2021). Most recently, all LGMDD1 disease-causing mutations were found to alter the structure of DNAJB6 such that the J-domain-G/F domain autoinhibitory mechanism is impaired (Abayev-Avraham et al., 2023). This leads to unregulated binding and hyperactivation of HSP70 in the absence of client protein, possibly trapping HSP70 in a non-productive interaction and leading to broader disruption of HSP70-dependent proteostasis (Box 1). Although an exact pathomechanism remains unknown, several lines of data now support an HSP70-dependent mechanism via either a dominant-negative or toxic gain of function (Abayev-Avraham et al., 2023; Bengoechea et al., 2015, 2020; Bhadra et al., 2022; Findlay et al., 2019, 2023; Kuiper et al., 2022; Sarparanta et al., 2012). To avoid potentially deleterious effects from complete DNAJB6 knockdown, a therapeutic approach that selectively targets the short DNAJB6b isoform was achieved using morpholinos, a type of ASO. Isoform specific knockdown of DNAJB6b resulted in partial normalization of a proteomic signature in cultured primary myotube from LGMDD1 mice (Table 2) (Findlay et al., 2023).

**Box 3. Clinical case presentation: an exemplary case of a dominantly inherited muscle disorder (Couthouis et al., 2014)**

The proband (iii-6; A, arrowhead) presented at 55 years of age to clinic, with a history of slow running and difficulty climbing stairs since the age of 12. He started using a cane at 37, two canes at 43 and, at 56 years of age, became wheelchair dependent. A bicep muscle biopsy at age 32 revealed myopathic changes with marked variation in fiber size and increased internal nuclei. There were also scattered fibers with rimmed vacuoles (B,C). Physical examination showed proximal predominant weakness involving shoulder abduction, elbow flexion, and hip flexion and extension. Distal strength was normal. An electrocardiogram and echocardiogram at age 54 were normal. He had mild exertional dyspnea with a forced vital capacity of 42% of predicted as well as sleep-disordered breathing, for which he required positive airway pressure support at night.

The proband's son (iv-6; A) was first evaluated by a neuromuscular specialist at 28 years of age owing to a history of slow running as early as age 8. By age 16, he had difficulty climbing stairs and rising from a sitting position. Physical examination revealed proximal greater than distal weakness affecting the lower extremities more than the upper, with prominent atrophy of the thigh adductors and the medial head of gastrocnemius, scapular winging and Achilles tendon contractures. He required the use of a cane at age 36. At age 37, he had no respiratory or cardiac symptoms, with a forced vital capacity of 90% of predicted, as well as a normal electrocardiogram and echocardiogram.

The proband's mother, brother, maternal grandmother and several others were similarly affected by progressive weakness (A). Genetic testing identified a c.265T>A mutation (p.Phe89Ile) in the *DNAJB6* gene, only in affected individuals, confirming a diagnosis of limb-girdle muscular dystrophy type D1-*DNAJB6* related (LGMDD1).

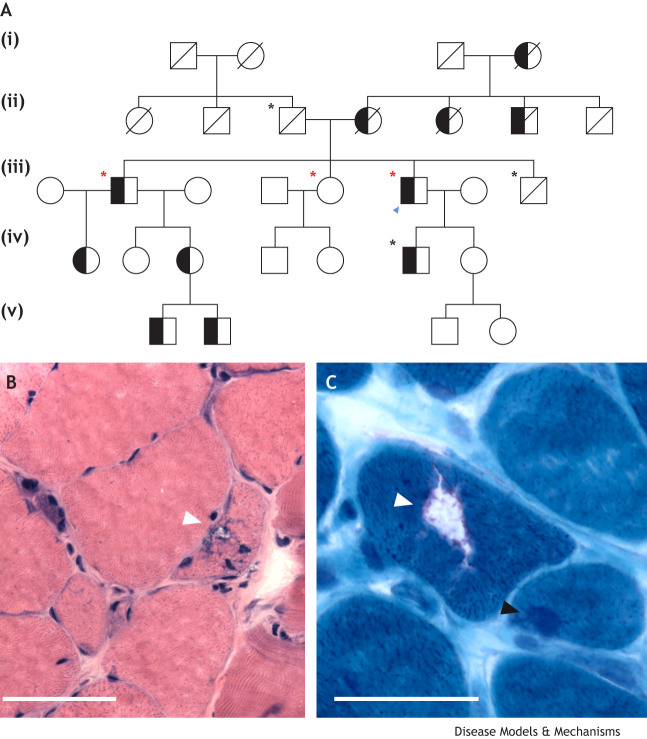
(A) Pedigree of a family with dominantly inherited LGMDD1. Exome sequencing was performed in individuals marked with a red asterisk and Sanger sequencing in those marked with a black asterisk. The proband is indicated by the blue arrowhead. Affected individuals are colored black and white to indicate dominant inheritance. (B,C) Histopathological examination of LGMDD1 muscle biopsy. (B) Photomicrograph of Hematoxylin and Eosin (H&E)-stained cryosection, showing evidence of an active myopathy with variation in fiber sizes, rounded atrophic fibers, an immature fiber with basophilic cytoplasm, and a fiber with rimmed vacuoles (white arrowhead) and internal nuclei. (C) Gomori trichrome-stained cryosection demonstrating a larger rimmed vacuole (white arrowhead) and a protein aggregate (black arrowhead). Scale bars: 50 μm.

Similar to DNAJB6, DNAJB4 is an HSP40 co-chaperone of HSP70, and a dominant point mutation (p.F90L) in the G/F domain of DNAJB4 was recently found to cause a late-onset distal myopathy with protein aggregate myopathology (Inoue et al., 2023). This residue corresponds to the F93 residue in its homolog, DNAJB6, which, when mutated (p.F93L, p.F93I), causes LGMDD1. Mice containing a knock-in p.F90L mutation in DNAJB4 develop a mild myopathy with protein aggregates in the soleus, where DNAJB4 expression is highest (Inoue et al., 2023). This F90L mutation enhanced TDP43 nuclear stress granule formation in an HSP70 interaction-dependent manner, suggesting a mechanism involving a gain of toxicity. Patients with recessively inherited *DNAJB4* mutations have been identified as well. They have a different phenotype with axial rigidity (Box 1), early respiratory failure, and muscle biopsies demonstrating similar features such as vacuoles and aggregates. *In vitro* studies indicate that recessive mutations result in reduced protein stability or a loss of function (Weihl et al., 2022). A DNAJB4 knockout mouse developed a similar phenotype with kyphosis (Box 1) and prominent diaphragm involvement, and similar muscle pathology to that seen in patients and the dominant p.F90L knock-in mouse. These results indicate that DNAJB4 is required for proper skeletal muscle function, leading the authors to suggest a dominant-negative mechanism for the heterozygous p.F90L mutation (Inoue et al., 2023; Weihl et al., 2022).

DNAJB2 is another HSP40 co-chaperone of HSP70 for which mutations are associated with neuromuscular disease (Sarparanta et al., 2023; Saveri et al., 2022). It has two isoforms produced via alternative splicing, which differ in their C-termini. The smaller DNAJB2A localizes diffusely and reduces TDP43 aggregation in an HSP70-dependent manner (Chen et al., 2016b). DNAJB2B primarily localizes to the cytoplasmic face of the ER and facilitates proteasomal degradation of ubiquitinated proteins via the ER-associated degradation (ERAD) pathway (Westhoff et al., 2005). DNAJB2 levels are highest in neurons, with DNAJB2B being the predominant isoform. Lower levels are found in skeletal muscle, with relatively similar levels of each isoform (Claeys et al., 2010). Recessive DNAJB2 mutations are associated with several axonal neuropathies, parkinsonian syndromes and a combined neuromyopathy with rimmed vacuolar pathology (Blumen et al., 2012; Gess et al., 2014; Liu et al., 2022; Sanchez et al., 2016). Recently, a dominantly inherited mutation causing a C-terminal extension of the DNAJB2A isoform c.832 T>G; p.(*278Glyext*83) was identified in a family with a late-onset neuromyopathy phenotype (Sarparanta et al., 2023). Mutant DNAJB2A has a transmembrane helix in the C-terminal extension, leading it to mislocalize to the ER and rapid degradation via the proteasome (Sarparanta et al., 2023). This also caused rapid degradation of co-expressed WT DNAJB2A, potentially explaining the reduced protein levels seen in patients' muscle biopsies (Sarparanta et al., 2023). The reduced expression of mutant DNAJB2A and the consequent increased turnover of WT DNAJB2A could support a dominant-negative mechanism of disease (Sarparanta et al., 2023). The extended mutant protein may also cause a toxic gain of function, similar to mutations causing a C-terminal extension of HSPB8.

### UPS and APL system

The UPS and APL system are both critical for maintaining skeletal muscle health, each playing unique and sometimes overlapping roles in protein quality control. The UPS facilitates the targeted degradation of misfolded or damaged proteins. Proteins destined for degradation are tagged with ubiquitin via a highly regulated process involving E1 ubiquitin-activating enzymes, E2 ubiquitin-conjugating enzymes and E3 ubiquitin ligases (Bedford et al., 2011). Once tagged, ubiquitinated proteins are degraded via the 26S proteasome, resulting in their breakdown into small peptides that can be recycled or further degraded (Bedford et al., 2011). The UPS plays a central role in muscle atrophy via degradation of myofibrillar proteins. Upregulation of UPS activity has been associated with muscle-wasting syndromes, such as sarcopenia and cachexia, whereas impaired UPS function can lead to myopathies with accumulations of damaged, misfolded proteins (Kitajima et al., 2020). Autophagy is similarly important for protein homeostasis within muscle, as it facilitates the turnover of long-lived proteins and damaged organelles, and responses to nutrient deprivation and starvation (Margeta, 2019; Xia et al., 2021). Targets of autophagy are sequestered in the cytoplasm within double-membraned vesicles called autophagosomes, which then fuse with lysosomes, enabling proteolysis of the engulfed cargo via their acidic pH. Mutations affecting autophagic and lysosomal proteins cause a variety of muscle disorders, commonly characterized by vacuolar histopathology (Margeta, 2019). There is evidence of crosstalk between the UPS and APL systems as dysfunction in one can lead to compensatory upregulation of the other (Kitajima et al., 2020).

*SQSTM1* encodes sequestesome-1, also known as P62, an adaptor protein with an autophagosome-interacting motif and a ubiquitin-associated (UBA) domain to facilitate interaction with ubiquitinated proteins. Like mutations in PrLDs of RBPs, mutations in proteins that facilitate ubiquitin-dependent autophagy, such as VCP and P62, also cause multisystem proteinopathy with several different phenotypes (FTD, ALS, PDB, rimmed vacuolar myopathy) (Ber et al., 2013; Bucelli et al., 2015; Fecto et al., 2011; Hocking et al., 2002; Watts et al., 2004). The relationship between these two classes of proteins (RBPs and ubiquitin-dependent autophagy) is illustrated by the process of autophagy-mediated stress granule clearance (Buchan et al., 2013; Seguin et al., 2014). Disease-causing mutations in P62 commonly impact its UBA domain and alter its ability to oligomerize and recruit ubiquitinated aggregates to autophagosomes, potentially leading to disease in vulnerable tissues by impairing the degradation and clearance of ubiquitinated aggregates, resulting in their toxic accumulation. A myopathy-associated mutation in *SQSTM1* (c.116511G>A) was initially described in a family with a dominantly inherited distal myopathy and one singleton case with a sporadic distal myopathy, both with rimmed vacuolar pathology (Bucelli et al., 2015). The mutation impacts splicing and leads to the generation of two abnormal P62 isoforms, one lacking its C-terminal UBA domain and another lacking its PEST2 domain, which has unclear functional significance but may impact protein stability (Bucelli et al., 2015; Rogers et al., 1986). When mutant P62 lacking its PEST2 domain is expressed in muscle, it aggregates in large inclusions (Bucelli et al., 2015). PEST2 domain missense mutations have been identified in FTD and ALS patients (Fecto et al., 2011; Rubino et al., 2012). The exact mechanistic category for *SQSTM1* mutations is unclear, but a toxic, gain-of-function mechanism has been suggested (Fecto et al., 2011). Some patients with a distal myopathy with rimmed vacuolar pathology resembling WDM but lacking disease-causing mutations in TIA1 were found to have a common TIA1 polymorphism (Lee et al., 2018). Further genetic testing revealed mutations in *SQSTM1* previously associated with Paget's disease of bone. Functional studies indicated that P62 and TIA1 similarly impact stress granule clearance. This interaction suggests a digenic mechanism, by which the TIA1 polymorphism dictates the tissue specificity associated with *SQSTM1* mutations. In a separate group of 50 patients with Paget's disease of bone with the same *SQSTM1* mutations, the TIA1 polymorphism was not present (Lee et al., 2018).

Valosin-containing protein (VCP) is a ubiquitously expressed, hexameric, ATPase associated with diverse cellular activities (AAA+). Dominant mutations in VCP cause MSPs, specifically, inclusion body myopathy, FTD, ALS and Paget's disease of bone, in a variety of combinations (Watts et al., 2004). Muscle histopathology is characterized by vacuoles, inclusions reactive for VCP, ubiquitin and TDP43, and tubulofilamentous inclusions as seen on electron microscopy (Weihl et al., 2009). VCP has several functions critical to protein quality control involving the UPS, autophagy regulation, stress granule homeostasis and many others (Meyer and Weihl, 2014). VCP acts as a ubiquitin-dependent segregase, extracting ubiquitinated proteins from multimeric complexes, and facilitates their degradation through the UPS or APL system (Ferrari et al., 2022; Meyer and Weihl, 2014; Taylor, 2015). Most VCP mutations increase ATPase activity and substrate processing, suggesting a gain-of-function mechanism (Blythe et al., 2017; Halawani et al., 2009; Weihl et al., 2006). However, some mutations fail to engage adaptor proteins necessary for VCP to function or are associated with reduced ATPase and unfoldase activity, suggesting a loss-of-function mechanism (Darwich et al., 2020; Ritz et al., 2011). Additionally, mutations with reduced ATPase activity are still capable of disrupting autophagosome maturation, suggesting a disconnect between cellular function and *in vitro* activity (Gonzalez et al., 2014; Wani et al., 2021; Weihl et al., 2015). In neurons, both conditional knockout of VCP and conditional expression of a dominant disease-associated mutation in a VCP-null background recapitulate features of FTD with TDP43 inclusions, suggesting a loss-of-function mechanism from hypomorphic VCP function (Wani et al., 2021). Several other studies have also suggested a loss-of-function and/or a dominant-negative effect regarding the impact of VCP on autophagy (Ferrari et al., 2022). Although an exact unifying mechanism for multisystem proteinopathy-causing VCP mutations is unclear, abnormal protein clearance is thought to be the key driver of pathology (Ferrari et al., 2022; Meyer and Weihl, 2014; Taylor, 2015). Recently, dominant mutations that cause VCP haploinsufficiency, or missense variants, the vast majority of which caused reduced ATPase activity, were found to be associated with developmental delay, intellectual disability, hypotonia and macrocephaly (Mah-Som et al., 2023). This childhood-onset neurodevelopmental syndrome and the associated mutations predicted to cause nonsense-mediated decay are distinct from VCP mutations associated with MSP, leading the authors to suggest differing pathomechanisms (Mah-Som et al., 2023).

Mutations in *LAMP2* cause Danon disease, an X-linked dominant disorder characterized by hypertrophic cardiomyopathy, skeletal myopathy and mental retardation in males, and a milder, primarily cardiac phenotype in females (Nishino et al., 2000). Myopathology is notable for cytoplasmic vacuoles filled with autophagic material and glycogen, and are lined by sarcolemmal proteins (sarcoglycans, dystrophin etc.), leading to their name, AVSF (Endo et al., 2015; Nishino et al., 2000). *LAMP2* encodes lysosome-associated membrane protein 2, a transmembrane protein localized to the limiting membrane of lysosomes. Three isoforms – LAMP2A, LAMP2B and LAMP2C – are created by alternative splicing of the terminal exon, each with varying roles in autophagy (Qiao et al., 2023). LAMP2A is ubiquitously expressed and acts as a receptor for the uptake of cytosolic proteins for lysosomal degradation in a process called chaperone-mediated autophagy (Qiao et al., 2023). LAMP2B is the predominant isoform in skeletal muscle, cardiac tissue and the brain, and is required for autophagosome-lysosome fusion (Konecki et al., 1995; Qiao et al., 2023). LAMP2C is involved in lysosomal-mediated degradation of RNA (RNautophagy) and DNA (DNautophagy) (Fujiwara et al., 2013a,b). Most *LAMP2* mutations associated with Danon disease cause a loss of function or expression of all LAMP2 isoforms, although some only affect LAMP2B, suggesting that this isoform is central to disease pathogenesis (Endo et al., 2015; Nishino et al., 2000; Qiao et al., 2023). Deficiency of LAMP2, specifically, LAMP2B, is now known to impair autophagosome-lysosome fusion, disrupting intracytoplasmic trafficking and macroautophagy (Chi et al., 2019). Further support for a loss-of-function mechanism involving LAMP2B was demonstrated by phenotypic improvement of *Lamp2* knockout mice, which lack all three isoforms and have key clinicopathological features of Danon disease, following gene-transfer treatment with AAV9-LAMP2B (Manso et al., 2020).

## Excitation-contraction coupling and Ca^2+^ handling

Excitation-contraction coupling is the physiological process of transforming sarcolemmal depolarization, an electrochemical signal, into mechanical force from the contraction of myofibrils. Following motor neuron activation, acetylcholine release into the synaptic cleft of the neuromuscular junction and motor endplate depolarization, an action potential travels along the sarcolemma and down into muscle via T-tubules. The triad complex (Box 1) is formed by the T-tubule and two flanking terminal cisterns of the sarcoplasmic reticulum, an intricate membranous network that surrounds myofibrils and stores, releases and re-uptakes calcium ions. The dihydropyridine receptor (DHPR) detects the action potential within the T-tubule and undergoes a conformational change, activating the ryanodine receptor (RYR1) within the terminal cistern of the sarcoplasmic reticulum. This leads to the release of calcium ions into the sarcoplasm, where they bind to troponin complexes, releasing the inhibition of actin-myosin cross-bridging and initiating muscle contraction. A variety of myopathies and muscular dystrophies can be caused by mutations in proteins critical for triad function and maintenance, leading to defects in excitation-contraction coupling and calcium homeostasis (Dowling et al., 2014, 2021; Jungbluth et al., 2018).

*RYR1* encodes the ryanodine receptor, a homo-tetrameric calcium channel that releases calcium from the sarcoplasmic reticulum as part of excitation-contraction coupling (Jungbluth et al., 2018; Zorzato et al., 1990). Mutations in *RYR1* cause a variety of myopathies that are broadly referred to as RYR1-related myopathies (Lawal et al., 2018, 2020). They were originally named based on their myopathological findings (central core disease, mini core myopathy, congenital fiber type disproportion, centronuclear myopathy) (Jungbluth et al., 2018; Klein et al., 2012; Lawal et al., 2020). Mutations in RYR1 causing reduced calcium release are generally associated with stable weakness as seen in congenital myopathies. This can result from recessive mutations causing reduced function or expression of RYR1. Dominant missense mutations are identified in ∼90% of central core disease patients, with some mutations suspected of having a dominant-negative effect on the WT protein (Avila and Dirksen, 2001; Fusto et al., 2022; Jokela et al., 2019; Klein et al., 2012; Loy et al., 2011; Monnier et al., 2001; SHY and MAGEE, 1956). Dominant mutations can also enhance calcium release via a gain-of-function mechanism and are typically associated with syndromes such as malignant hyperthermia, exertional rhabdomyolysis and others (Dlamini et al., 2013; MacLennan et al., 1990). An allele-specific knockdown method, involving the local *in vivo* delivery of siRNA directly into the footpad muscles of RYR1 mouse models of malignant hyperthermia with cores and central core disease, enhanced overall RYR1 function (Table 2) (Loy et al., 2012). More recently, a CRISPR-Cas9 approach was used to specifically inactivate the mutated *RYR1* allele by targeting frequent single-nucleotide polymorphisms (SNPs) segregating on the same chromosome (Table 2) (Beaufils et al., 2024).

*DNM2* encodes the protein dynamin 2, a ubiquitously expressed GTPase with many functions, including membrane trafficking and fission via its pinch-ase activity (Fujise et al., 2021; Liu et al., 2011; McNiven, 1998). Dominant *DNM2* mutations cause a centronuclear myopathy that is clinically characterized by progressive distal and proximal weakness beginning during infancy or even young adulthood, as well as ptosis, ophthalmoparesis, facial weakness, dysphagia and respiratory insufficiency (Bitoun et al., 2005; Hayes et al., 2022; Spiro et al., 1966). Compared to other centronuclear myopathies, those due to dominant *DNM2* mutations tend to be less clinically severe (Jungbluth et al., 2018). Myopathology demonstrates prominent nuclei in the middle of myofibers, myofiber hypotrophy, organelle disorganization and an absence of ongoing myofiber degeneration (Hayes et al., 2022). Another key feature of centronuclear myopathies is abnormal formation or maintenance of the triad complex structure, resulting in impaired excitation-contraction coupling and weakness (Al-Qusairi and Laporte, 2011; Dowling et al., 2014, 2021). Most *DNM2* mutations associated with centronuclear myopathy lead to reduced inhibition of its GTPase activity (Hayes et al., 2022). Reducing dynamin 2 activity by various methodologies can rescue mouse model disease phenotypes, supporting the hypothesis that increased dynamin 2 activity is the common disease pathomechanism (Buono et al., 2018; Dowling et al., 2021; Hayes et al., 2022; Rabai et al., 2019; Trochet et al., 2018).

LGMDR1 (LGMD2A) is caused by recessive mutations in calpain-3 (encoded by *CAPN3*), a calcium-dependent cysteine protease that functions as a homodimer and is anchored to the giant protein titin at its N2A and M lines (Dowling et al., 2021). It plays an important role in sarcomere maintenance by proteolytically cleaving proteins (titin, filamin C, myosin light chain 2), leading to their degradation via the proteasome (Cohen et al., 2006; Guyon et al., 2003; Kramerova et al., 2007). At titin's N2A line, where the T-tubule enters the myofibril, calpain-3 stabilizes the triad complex (one T-tubule and two adjoining terminal cisterns of sarcoplasmic reticulum) via its interaction with RYR1 (Dowling et al., 2021; Kramerova et al., 2007). LGMDR1 mutations cause loss of expression or impair calpain-3 proteolytic activity, anchorage to titin or ability to homodimerize (Dowling et al., 2021; Ermolova et al., 2011; Ono et al., 1998; Richard et al., 1999). Dominantly inherited mutations in *CAPN3*, specifically in-frame deletions and point mutations, were found to cause a more mild LGMD phenotype, which is now classified as LGMDD4 (Angelini, 2020; Vissing et al., 2016). These mutations are suspected to have a dominant-negative effect, although an exact, unifying mechanism remains unknown (Cerino et al., 2020; González-Mera et al., 2021; Vissing et al., 2016).

*ORAI1* encodes calcium release-activated calcium channel (CRAC) protein 1, an ion channel that facilitates extracellular calcium entry into the sarcoplasm following calcium depletion from the sarcoplasmic reticulum (Nesin et al., 2014). This process is known as store-operated calcium entry (SOCE; Box 1). Dominant gain-of-function mutations that lead to increased calcium entry cause tubular aggregate myopathy (TAM) type 2 (Nesin et al., 2014).

*STIM1* encodes stromal interacting molecule 1, a calcium-sensitive activator of the calcium channel encoded by *ORAI1* (Böhm et al., 2013; Nesin et al., 2014). Dominant gain-of-function mutations in *STIM1* lead to constitutive activation of CRAC protein 1, overactive SOCE and are associated with TAM type 1 (Böhm et al., 2013; Nesin et al., 2014).

Dominantly inherited mutations in other genes involved in excitation-contraction coupling and calcium handling, such as *CASQ1* and *CACNA1S*, have also been linked to various disorders of skeletal muscle and are described in Table 1 (Rossi et al., 2014, 2022; Schartner et al., 2017).

## Membrane repair

Muscle contraction generates high forces on the sarcolemma, creating small tears in the membrane. This causes localized calcium entry and a subsequent cascade of cellular repair events, somewhat analogous to the mechanisms of neurotransmitter release, by which membranous vesicles are trafficked to and fuse with the injury site, thus sealing the tear (Demonbreun et al., 2016; Dowling et al., 2021).

*DYSF* encodes dysferlin, a sarcolemmal protein that plays a key role in skeletal muscle membrane repair. Recessive loss-of-function mutations in dysferlin cause LGMD R2. Recently, a large family with a late-onset muscular dystrophy was found to have a dominantly inherited frameshift mutation affecting the terminal exons of DYSF. The exact mechanism is not known, but the mutant transcript appears to evade nonsense-mediated decay, and the authors suggest that the truncated dysferlin could have a dominant-negative impact (Folland et al., 2022).

*CAV3* encodes caveolin-3, a muscle-specific structural protein within caveolae, a special type of lipid raft formed by small infoldings of the plasma membrane, which play a role in endocytic vesicles and signal transduction (Betz et al., 2001; Minetti et al., 1998). Mutations in *CAV3* have been associated with several phenotypes, including rippling muscle disease, LGMD and hyperCKemia, and are thought to cause disease via a dominant-negative mechanism involving retention and aggregation of normal caveolin-3 within the Golgi complex (Galbiati et al., 1999; Herrmann et al., 2000).

## Metabolic

Metabolic myopathies can be caused by abnormalities in fatty acid transport and oxidation (fatty acid oxidation disorders), glycogenolysis/glycolysis (glycogen storage disorders; Box 1) and mitochondrial metabolism (mitochondrial myopathies) (Tarnopolsky, 2022). Glycogen storage disorders often manifest during brief bouts of high-intensity exercise, while mitochondrial myopathies and fatty acid oxidation disorders commonly manifest during long-duration endurance activities in combination with metabolic stressors, such as fasting. They can present neonatally with hypotonia and encephalopathy, or during childhood or young adulthood with exercise intolerance, rhabdomyolysis and weakness (Tarnopolsky, 2022).

*PYGM* encodes myophosphorylase, a muscle-specific glycogen phosphorylase that breaks down glycogen for use by muscle. Recessive mutations cause the glycogen storage disorder, McArdle's disease, which causes painful muscle cramps and weakness during exertion (Lebo et al., 1984; McArdle, 1951). Dominant missense *PYGM* mutations were identified in a family with adult-onset weakness with glycogen accumulation and aggregates on muscle biopsy (Echaniz-Laguna et al., 2020). The mechanism is unknown, but the mutant myophosphorylase aggregates with desmin (Echaniz-Laguna et al., 2020).

*MB* encodes myoglobin (Box 1), a protein located primarily in skeletal and cardiac muscle fibers that reversibly binds oxygen via its heme prosthetic group. It has a higher affinity for oxygen than hemoglobin, allowing it to effectively act as an oxygen storage protein, binding and releasing oxygen depending on its concentration gradient. Several families with a dominantly inherited myopathy characterized by proximal and axial predominant weakness that progresses to involve distal muscles, as well as cardiac and respiratory involvement, were found to have a point mutation within *MB* (Olivé et al., 2019). Muscle histopathology was notable for vacuoles and sarcoplasmic inclusions. The mutant myoglobin was found to have altered O_2_ binding and was suspected of causing disease via a gain of function related to elevated superoxide levels (Olivé et al., 2019).

## Conclusions

Similar to recessive muscle disorders, dominantly inherited myopathies and muscular dystrophies arise from mutations that affect a broad spectrum of skeletal muscle functions, ranging from key structural elements of myofibers, spanning the ECM to the nuclear envelope, to protein homeostasis mechanisms. Some mechanisms, such as those involving repeat expansions or mutations in the PrLDs of RBPs, are inherited either exclusively or predominantly in a dominant fashion. Apart from these particular cases, dominant mutations affect many of the same proteins as those involved in recessive disorders of muscle. However, their pathomechanisms are distinct from the loss of function or expression seen in recessive conditions.

Although significant progress has been made for some disorders, a better understanding of the complex, heterogeneous, disease mechanisms underlying dominantly inherited myopathies and muscular dystrophies is a key unmet need necessary for therapeutic development. Haploinsufficiency, dominant-negative, toxic and gain-of-function mechanisms are helpful categories for conceptualizing mechanisms of dominance and guiding therapeutic strategy development. However, these are often oversimplifications, as many disorders do not neatly fit into a category, as mutant proteins may display a combination of toxicity, loss of some functions, and gain of others. For disorders due to dominant-negative, toxic and/or gain-of-function mechanisms, understanding tissue-specific tolerances to gene knockout and haploinsufficiency will enable the design of effective and safe knockdown therapeutic approaches. Conversely, the possibility of toxic overexpression of WT protein is a concern for gene-replacement therapies for haploinsufficiency disorders, as well as knockdown-and-replace strategies used for other dominant disorders. The field of gene therapy still faces several significant hurdles, such as dealing with pre-existing antibodies to AAV, navigating the potentially lethal risks tied to immune reactions, and addressing how the healthcare system will manage the high costs of gene therapies for the numerous individuals with rare diseases who could benefit. Lastly, as RNAi- and ASO-based therapies are entering clinical use, with ongoing advancements in their delivery to skeletal muscle, and further characterization of natural histories for clinical trial preparedness, we will have the necessary tools to develop and evaluate treatments for these rare dominantly inherited muscle disorders.
